# An improved method to detect arrhythmia using ensemble learning-based model in multi lead electrocardiogram (ECG)

**DOI:** 10.1371/journal.pone.0297551

**Published:** 2024-04-09

**Authors:** Satria Mandala, Ardian Rizal, Adiwijaya, Siti Nurmaini, Sabilla Suci Amini, Gabriel Almayda Sudarisman, Yuan Wen Hau, Abdul Hanan Abdullah

**Affiliations:** 1 Human Centric (HUMIC) Engineering, Telkom University, Bandung, Indonesia; 2 School of Computing, Telkom University, Bandung, Indonesia; 3 Intelligent System Research Group, Universitas Sriwijaya, Palembang, South Sumatra, Indonesia; 4 Department of Cardiology and Vascular Medicine, Faculty of Medicine, Universitas Brawijaya, Malang, East Java, Indonesia; 5 IJN-UTM Cardiovascular Engineering Centre, Faculty of Electrical Engineering, Universiti Teknologi Malaysia, Johor Bahru, Johor, Malaysia; 6 Faculty of Computing, Universiti Teknologi Malaysia, Johor Bahru, Johor, Malaysia; Menoufia University, EGYPT

## Abstract

Arrhythmia is a life-threatening cardiac condition characterized by irregular heart rhythm. Early and accurate detection is crucial for effective treatment. However, single-lead electrocardiogram (ECG) methods have limited sensitivity and specificity. This study propose an improved ensemble learning approach for arrhythmia detection using multi-lead ECG data. Proposed method, based on a boosting algorithm, namely Fine Tuned Boosting (FTBO) model detects multiple arrhythmia classes. For the feature extraction, introduce a new technique that utilizes a sliding window with a window size of 5 R-peaks. This study compared it with other models, including bagging and stacking, and assessed the impact of parameter tuning. Rigorous experiments on the MIT-BIH arrhythmia database focused on Premature Ventricular Contraction (PVC), Atrial Premature Contraction (PAC), and Atrial Fibrillation (AF) have been performed. The results showed that the proposed method achieved high sensitivity, specificity, and accuracy for all three classes of arrhythmia. It accurately detected Atrial Fibrillation (AF) with 100% sensitivity and specificity. For Premature Ventricular Contraction (PVC) detection, it achieved 99% sensitivity and specificity in both leads. Similarly, for Atrial Premature Contraction (PAC) detection, proposed method achieved almost 96% sensitivity and specificity in both leads. The proposed method shows great potential for early arrhythmia detection using multi-lead ECG data.

## Section 1: Introduction

Cardiovascular disease (CVD) is the leading cause of death globally, resulting in 17.9 million deaths in 2019. Among these fatalities, heart attacks and strokes account for 85% of the total [1]. Notably, arrhythmia, characterized by abnormal heart rhythms, possess the potential to serve as triggers for CVD. Arrhythmia can arise from diverse factors, encompassing heart disease, electrolyte imbalances, and medication effects [2].

Arrhythmia causes several distinct classes of diseases, classified based on variations in electrical impulses. Among them, Atrial Fibrillation (AF) stands as one of the most prevalent arrhythmia, characterized by rapid and irregular atrial rhythms that can precipitate strokes [3]. Additionally, Premature Atrial Contraction (PAC) represents another form, defined by the premature contraction of the atria due to aberrant electrical impulses [4]. Furthermore, Premature Ventricular Contraction (PVC) arises when the ventricles undergo premature depolarization, leading to potential complications such as chest pain and premature cardiac failure [5].

Typically, an arrhythmia is detected through the utilization of ECG which serves as a diagnostic tool for recording the electrical activity of a patient’s heart. Compared to alternative techniques, the ECG offers the advantage of non-invasiveness and ease of operation [6]. The ECG can be performed using a single lead or up to 12 leads, with each lead representing a specific electrode placement to capture the heart’s electrical signals [7]. The ECG signal exhibits three primary waveforms, namely P waves, QRS complexes, and T waves, which can be employed as markers for identifying an arrhythmia [8].

Ensemble learning techniques, such as boosting, bagging, and stacking, have witnessed significant advancements in recent years for the classification of arrhythmia based on ECG signals. While many studies explored these methods for single-lead arrhythmia detection, research on applying ensemble learning to multi-lead arrhythmia classification was relatively scarce. A limited number of studies, including [9–16], contributed to this area. Lee et al. [9] introduced a feature extraction method and evaluated multiple classifiers, including XGBoost, achieving 90.46% accuracy and 89.2% sensitivity. Similarly, Ye et al. [10]. utilized XGBoost, CNN, and BiLSTM for 12-lead ECG classification, with an accuracy of 96.4% and sensitivity of 78.8%. Zheng et al. [11] proposed a multi-stage approach with XGBoost, resulting in 99.2% accuracy. Other ensemble algorithms, such as bagging decision trees in studies by Mert et al. [12] and Afkhami et al. [13], achieved accuracies of 99.51% and 99.70%, respectively. Jadhav et al. [14] used the Random Subspace Ensemble Classifier with an accuracy of 91.11%. On the other hand, Zhou et al. [15] introduced an ensemble deep learning method combining CNN and LSTM for premature ventricular contraction (PVC) classification.

In recent years, the landscape of arrhythmia classification has remained limited. Besides that, a critical examination of existing studies reveals several noteworthy issues, especially in the context of boosting algorithms. The identified problem lies in the lack of emphasis on specificity metrics in prior investigations, and despite achieving high accuracy levels, sensitivity performance often falls short of optimal standards. Moreover, the presentation of results in previous studies tends to be simplistic, raising concerns about the viability of multi-lead approaches in arrhythmia research. The identified issues underscore the necessity for an improved method that not only enhances sensitivity and specificity metrics but also ensures a comprehensive evaluation of performance. Additionally, the presentation of performance results for the existing studies was often simplistic, which raised concerns regarding the utilization of multi-lead approaches in the research.

Based on the problems above, this study introduces a comprehensive investigation into multi-class arrhythmia detection using an ensemble learning approach, specifically the Fine Tuned Boosting (FTBO) model. The primary contributions of this research can be outlined as follows:

- Introduction of FTBO Model: Proposes a novel ensemble learning model, the Fine Tuned Boosting (FTBO), for enhanced multi-class arrhythmia detection in 2-lead ECG signals.
- Innovative Feature Extraction Technique: Introduces a new feature extraction technique involving dynamic features, namely RR interval and QRS complex, over a time span of 1 to 8 hours. Utilizes a sliding window with a window size of 5 R-peaks, deviating from traditional approaches.
- Addressing Specificity Metrics in Boosting Algorithms: Recognizes and addresses the infrequent reporting of specificity metrics in boosting algorithm studies, aiming to provide a more comprehensive evaluation of the proposed model’s performance.
- Comparative Analysis: Compares the performance of the FTBO model with other ensemble models commonly used in arrhythmia detection. This comparative analysis aims to highlight the strengths and potential improvements of the proposed model.
- Parameter Tuning Exploration: Investigates the impact of adjusting parameters on the performance of the FTBO model. This exploration seeks to optimize the model’s sensitivity and specificity for more robust arrhythmia detection.

Collectively, these contributions aim to advance the field of arrhythmia detection in multi-lead ECG signals, addressing previous limitations and providing a foundation for improved accuracy and applicability in real-world healthcare scenarios.

In the subsequent sections, this research unfolds in a structured manner. Section 1 serves as the introduction, providing a comprehensive overview of the motivation, objectives, and challenges addressed by this study. Section 2 conducts a thorough review of related works. In Section 3, the materials and methods used are carefully described. In Section 4, the proposed approach delves into the Fine-Tuned Boosting (FTBO) model, the new feature extraction method, and the dataset that was used to find multiple types of arrhythmia in multi-lead ECG signals. Section 5 presents the experimental results, showcasing the performance of the FTBO model compared to other ensemble models and exploring the impact of parameter tuning. Following this, Section 6 engages in a comprehensive discussion of the obtained results, their implications, and the broader context of the study within the field. Section 7 draws conclusions, summarizing key findings, highlighting contributions, and paving the way for potential future research directions.

## Section 2: Related works

Numerous studies were conducted to investigate the application of ensemble learning classifiers for arrhythmia detection, as evidenced by works such as [12, 17–21]. These studies explored the effectiveness of three main ensemble learning methods: bagging, stacking, and boosting. Furthermore, alternative ensemble classifiers, including multi-layer perceptron and random forest, were also proposed for arrhythmia detection, as documented in [22, 23]

Researchers have extensively explored the bagging technique for arrhythmia detection, showcasing its versatility and effectiveness in various contexts. Zeng et al. [24] introduced the Selection Base Classifier on Bagging (SBCB) method as a potent approach in this domain. Building on this, Mert et al. [12] and Afkhami et al. [13] harnessed the Bagging Decision Tree (BDT) algorithm on ECG signals obtained from two leads, showcasing its efficacy in arrhythmia detection. Rizwan et al. [19] employed the Uniform Directional Binary (UDB) technique for arrhythmia detection, focusing exclusively on single-lead ECG signals. Bilgin et al. [25] utilized bagging bootstrap aggregation as a method to detect a specific type of arrhythmia, namely Paroxysmal Atrial Fibrillation (PAF), associated with an increased risk of stroke. Following this, Plawiak et al. [26] extended the bagging technique by employing two-layer classifiers, specifically Support Vector Machine (SVM) models of nu-SVC and C-SVC linear types, applied to single lead ECG Modified Limb lead II (MLII) signals. Finally, Hussain et al. [27] further investigated the bagging technique, considering three distinct classifiers: ensemble bagged tree, subspace tree, and Random Undersample (RUS) boosted tree for effective arrhythmia detection. This chronological arrangement emphasizes the evolution of bagging techniques in the context of arrhythmia detection.

In the context of arrhythmia detection, the stacking technique has been explored by several researchers. Warrick et al. [17, 28] were pioneers in proposing the CL3 algorithm, a fusion of Convolutional Neural Network (CNN) and Long-Short Term Memory (LSTM). This algorithm was intricately designed for efficient arrhythmia detection in single-lead ECG signals, employing a stacking framework to enhance overall performance. Following this, Nandhini et al. [29] suggested a stacking technique for detecting arrhythmia. They used several base learners such as Linear Discriminant Analysis (LDA), K-Nearest Neighbor (KNN), Support Vector Machine (SVM), Classification and Regression Tree (CART), and Naive Bayes. The meta-learner utilized in the study was the Random Forest (RF) algorithm. Most recently, Essa et al. [21] developed an arrhythmia detector based on the stacking technique, utilizing a Deep Learning Bagging Model as the base learner. The meta-learner in this study was a combination of CNN-LSTM and RRHOS-LSTM models, highlighting the versatility of stacking in combining deep learning architectures. This chronological review underscores the evolution and continued relevance of the stacking technique in the context of arrhythmia detection.

The following studies demonstrated the benefits of boosting techniques for improving arrhythmia detection accuracy and highlighted the promise of different algorithms for further improving accuracy. Hong et al. [18] proposed a boosting technique using the XGBoost classification algorithm for the detection of arrhythmia in single lead ECG signals. The results obtained by Hong et al. were highly satisfactory, achieving a maximum F1-score of 86%. Similarly, Yue et al. [20] also reported satisfactory results using the same method, with accuracy values and F1-scores of 86% and 84%, respectively. Peimankar et al. [30] employed a boosting technique for arrhythmia detection, utilizing four classification algorithms: Random Forest, Adaboost, Artificial Neural Network (ANN), and Dempster-Shafer Combination Rule. The performance of these algorithms was evaluated using a 5-fold cross-validation technique, yielding impressive results. The study reported a sensitivity of 90.37%, specificity of 97.62%, and accuracy of 96.18%. However, the authors noted that their detection performance was slightly lower compared to similar studies. Several other studies also explored the use of boosting techniques for arrhythmia detection in single lead ECG. Mahmood et al. [31] focused on detecting Atrial Fibrillation (AF) arrhythmia and compared the performance of multiple machine learning algorithms, including Decision Tree, Random Forest, SVM, and KNN. The AdaBoost Ensemble classifier demonstrated the best performance with an accuracy of 97.4%, outperforming the other algorithms. Ganapathy [32] achieved a relatively high-performance value of 98.3% using AdaBoost. In addition, Ketu et al. [33] incorporated the Synthetic Minority Oversampling Technique (SMOTE) to balance the data in their research, resulting in the highest accuracy of 99.9%.

The following studies used ensemble learning and various classification algorithms to accurately detect arrhythmia using ECG signals. Jadhav et al. [14] utilized a Partial Decision Tree (PART) as a base classifier, combined with the Random Subspace Ensemble Classifier, for arrhythmia detection. Kim et al. [34] proposed a novel classification method for arrhythmia detection based on Ensemble Learning and the Taguchi Method. Sultan et al. [35] focused on arrhythmia detection in single-lead ECG and employed an ensemble method based on Decision Trees as the classifier. Zhou et al. [15] used a combination of Lead Convolutional Neural Network (LCNN) and Long-Short Term Memory (LSTM) to detect Premature Ventricular Contraction (PVC) arrhythmia in single lead ECG. Manju et al. [36] conducted an arrhythmia detection study using 12 leads and employed the SMOTEENN technique for data balancing. XGBoost was used for feature extraction, and four classification algorithms (Decision Tree, Random Forest, KNN, and SVM) were employed. The experimental results demonstrated that SVM outperformed the other three algorithms, achieving an accuracy of 97.35%. Singh et al. [1] conducted a study comparing classification algorithms, including Linear SVM, Random Forest, and JRipper (JRip), for arrhythmia detection. Ihsanto et al. [37] introduced an arrhythmia detection method using Depthwise Separable Convolutional, All Convolutional Network (ACN), Batch Normalization, and CNN classification techniques. Wu et al. [38] conducted a study on arrhythmia detection in single lead ECG using three ensemble learning algorithms: Bagging, Stacking, and Boosting (AdaBoost). Four machine learning algorithms, namely Decision Tree, KNN, SVM, and ANN, were employed as base algorithms in the bagging and stacking techniques. The study employed 10-fold cross-validation to evaluate the performance of these ensemble techniques. The stacking technique achieved the highest accuracy of 92%, followed by bagging with 89% and boosting with 88%. On the other hand, Dalal et al. [22] and Yakut et al. [23] proposed arrhythmia detection methods based on the ensemble of Multi-Layer Perceptron and Random Forest. One distinction between the two studies is the use of meta-learners, where Yakut et al. [23] incorporated a meta-learner while Dalal et al. [22] did not.

In line with the previous discussion, three studies focused on features exploration in detecting arrhythmia, specifically Atrial Fibrillation (AF) and Ventricular Arrhythmia (VArr) are explained in [39–41]. The first study by Plesinger et al. [39] conducted 43 QRS and PQRS features to identify AF on Single-lead Holter ECG readings. The classification technique involved bagging with the Bagged Tree Ensemble algorithm, comprising a Simple Decision Tree, Shallow Neural Network, and Support Vector Machine (SVM). The study reported lower sensitivity, specificity, and accuracy values of 74%, 80%, and 82% respectively compared to the performance achieved by the CNN algorithm. The presence of noise in the dataset could explain the disparity in results. The second study by Shi et al. [40] conducted a research study that emphasized the utilization of a diverse range of features extracted through Discrete Wavelet Transform (DWT) and Principal Component Analysis (PCA) for the analysis of single lead ECG data. The ensemble technique employed in this study was stacking, which combined multiple base learners, including K-Nearest Neighbors (KNN), Support Vector Machine (SVM), Decision Tree, and Random Forest. The outputs from the base learners were then fed into a meta-learner that utilized the SVM algorithm. The selection of SVM as the secondary classifier or meta-learner was driven by the limited size of the dataset and the features employed. The study specifically focused on analyzing single lead ECG data, particularly MLII. The research achieved an accuracy of 74.5%. The third study by Rezaei et al. [41] utilized various features, such as R-R Interval and QRS Duration, and employed a boosting-based classifier (XGBoost with a decision tree). The performance of the model was evaluated through rigorous 10-fold cross-validation, resulting in remarkable accuracy, sensitivity, and specificity values of 99%, 98.6%, and 90.95% respectively. These results surpassed previous studies in the field, indicating the effectiveness of the proposed approach. It is important to note that the accuracy value is relatively lower compared to previous studies on Inter-Patient analysis conducted by De et al. [42], Luz et al. [43], Li et al. [44], and Chen et al. [45].

## Section 3: Materials and methods

### Section 3.1: Materials

#### Section 3.1.1: Data

The data used in this study is data from the MIT-BIH Arrhythmia Database as described in [11–13, 15, 46–48]. The data will be used to analyze and detect three types of arrhythmia: AF, PVC, and PAC. Details about the data are as follows: 22 records are AF signals, and 47 are PVC and PAC signals, respectively. The record length of each data varies in the range of 1 hour to 8 hours.

#### Section 3.1.2: Environment

In developing this research, hardware and software were used as the main resources. For hardware, computers or laptops serve as the primary tools for running the utilized software [49].

Meanwhile, the software used is the Python programming language for conducting data analysis and data processing. Additionally, several digital platforms are implemented for data visualization [50].

### Section 3.2: Methods

#### Section 3.2.1: Research scenario

The determination of three distinct scenarios in this study is grounded in the pursuit of a comprehensive understanding of machine learning models for arrhythmia detection. Each scenario serves a specific investigative purpose, collectively contributing to a nuanced evaluation of model performance. The first scenario, focusing on the FTBO model, explores the impact of hyperparameter tuning on its efficacy in detecting various arrhythmia classes. This attention to hyperparameter optimization is pivotal for refining the model’s sensitivity to subtle patterns in the data. In the second scenario, this study is expanded to include both classical machine learning models and ensemble learning models. The goal is to find the best learning model for detecting different types of arrhythmia. This diversification ensures a holistic exploration of model capabilities. In the third scenario, the analysis is expanded to include other ensemble learning models, such as bagging and stacking. This adds a comparison part to find the best ensemble model for finding arrhythmias. The combination of these scenarios is strategically designed to yield comprehensive insights into the strengths, weaknesses, and optimal configurations of machine learning models in the context of arrhythmia detection.

- First Scenario
  In the first scenario, compare the performance of the boosting algorithm before hyperparameter tuning and after hyperparameter tuning (referred to as FTBO). This scenario is applicable to all arrhythmia classes, including AF, PVC, and PAC. Additionally, cross-validation is implemented during model training to optimize overall model performance.
- Second Scenario
  In addition to employing ensemble learning models, this research also conducted tests on classical machine learning models for the detection of AF, PVC, and PAC. Three classical machine learning models were used: decision tree, SVM, and Logistic regression. The objective was to determine which learning model is most effective in detecting the three classes of arrhythmia. In this second scenario, cross-validation is also applied to enhance overall performance.
- Third Scenario
  In the third scenario, this study conducted other ensemble learning models, specifically the bagging and stacking models. Similar to the first scenario, hyperparameter tuning was implemented for both of these ensemble models to find the optimal parameters. Additionally, cross-validation was applied to optimize model performance. Subsequently, this study compared the performance of these two models with the boosting model from the first scenario. This comparison aims to see which ensemble model effectively detecting the three arrhythmia classes: AF, PVC, and PAC.

#### Section 3.2.2: Comparison

Four comparisons were conducted to achieve optimal results. First, a comparison was made based on the performance results of the first scenario to assess the default performance of the ensemble boosting model and after hyperparameter tuning (FTBO).Second, the performance results from the second scenario were compared with the result of the first scenario. This comparison presented the performance outcomes of three classical machine learning algorithms and the boosting model.

Third, comparisons were performed on the results of the third scenario, where the performance of the bagging and stacking ensemble models was compared with that of the boosting model in the first scenario. Finally, this study compared its results with several previous studies that focused on arrhythmia detection using ECG signals. This comparison aims to evaluate the effectiveness of the proposed method.

## Section 4: Proposed method

**Fig 1:**
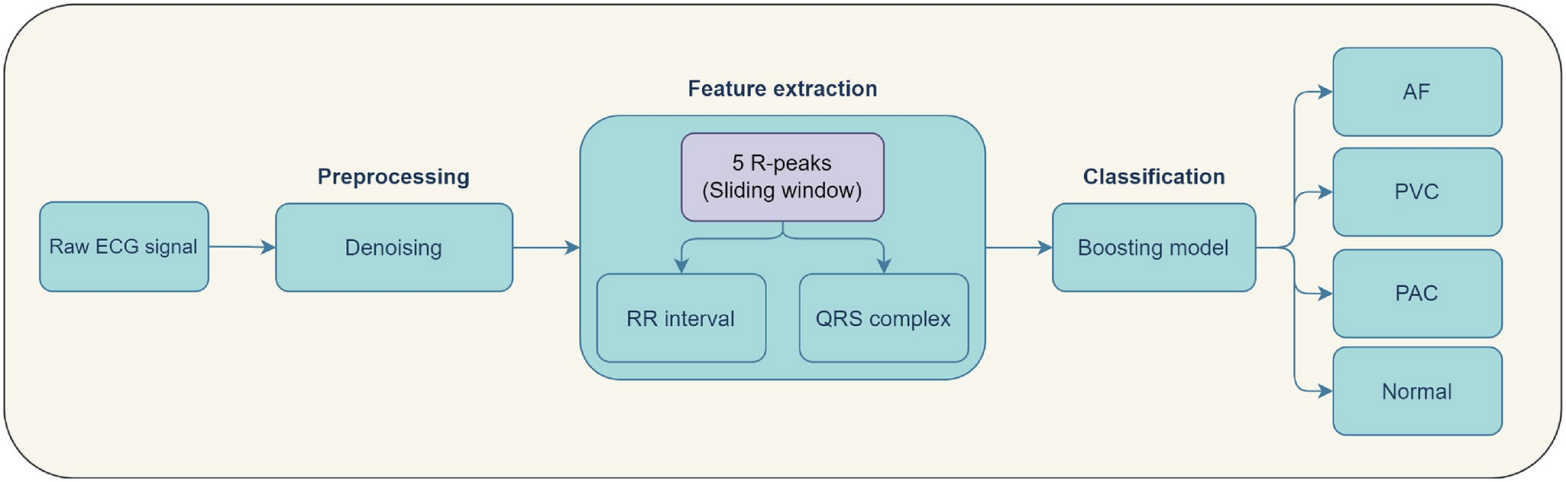
Proposed method to detect arrhythmia.

This study proposes a new method to detect three classes of arrhythmias: AF, PVC, and PAC in multi-lead ECG signals. The proposed method includes several processes, namely denoising, feature extraction, and classification. Furthermore, the proposed method is shown in Fig 1.

Adaptive filters used to remove noise from signals in the preprocessing stage. Moreover, at the feature extraction stage this study propose a new technique, namely implementing a sliding window for each of the 5 R peaks to be extracted into QRS complex and RR interval. Finally, this research proposes the Fine Tuned Boosting (FTBO) model as a classification model. Furthermore, a more detailed explanation can be seen in the following sections.

### Section 4.1: Preprocessing

**Fig 2:**
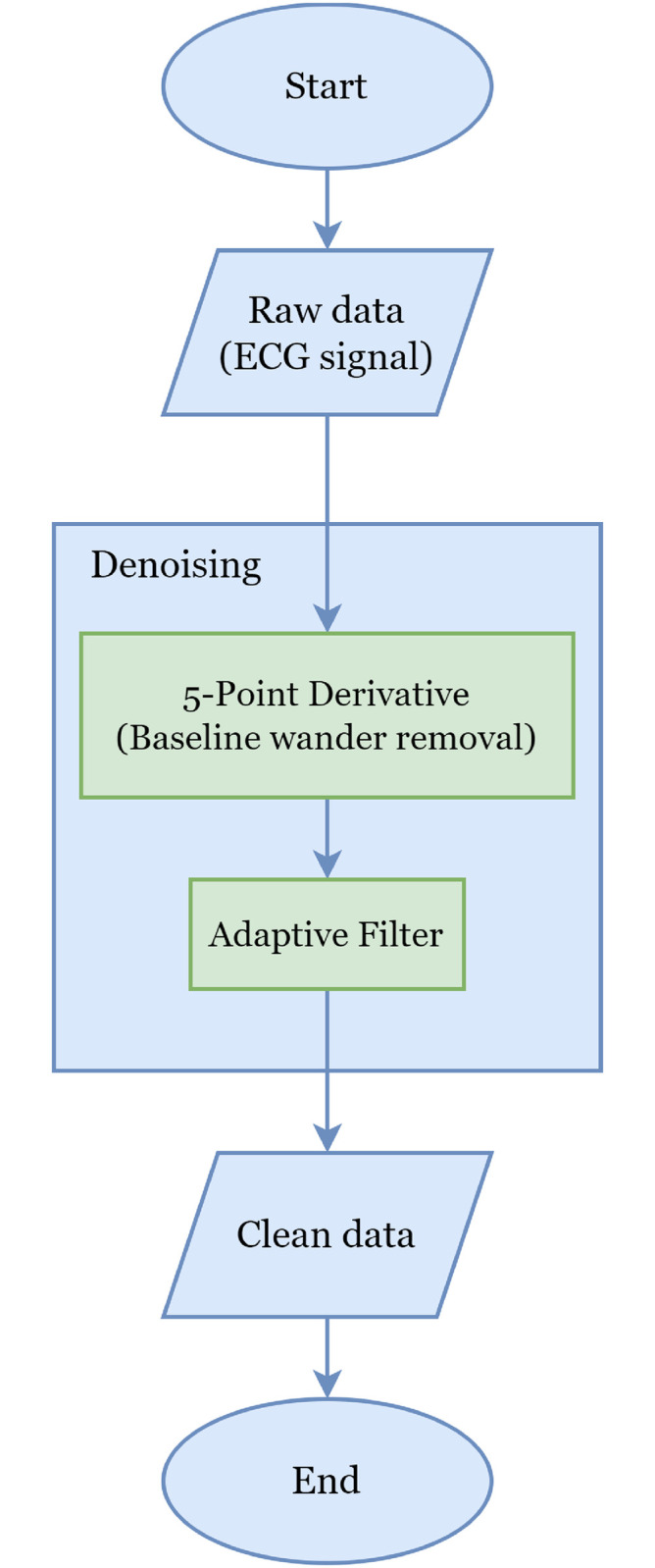
Preprocessing raw signal ECG.

Fig 2 is an illustration of the data pre-processing process in this study. The denoising process is used to remove noise in the raw ECG signal. This denoising process uses the adaptive filter method and 5-point Derivative to remove the baseline wander on a [51] signal.

### Section 4.2: Feature extraction

**Fig 3:**
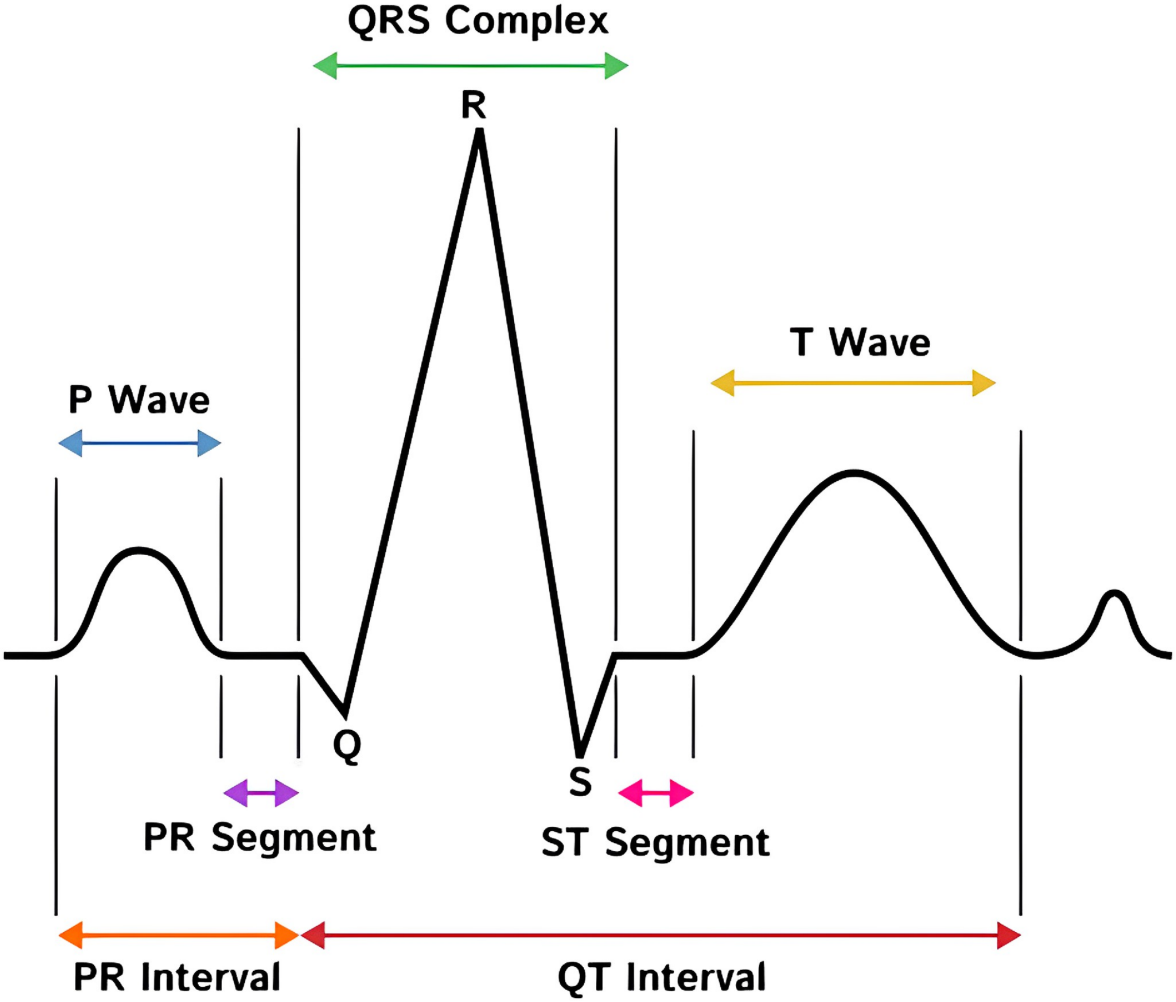
ECG waves.

Fig 3 illustrates a typical ECG signal waveform. As shown in Fig 3, the ECG signal has three fundamental waves, namely P, QRS Complex, and T. Several characteristics can be extracted from the ECG signal to determine the type of arrhythmia based on the dynamic features of the ECG signal [52]. However, this study only uses two dynamic features of the ECG signal, namely the R-R interval and the width of the QRS Complex signal. The R-R interval is the interval between the R wave and the next R wave with a duration between 0.6–1.2 seconds (s) [53]. At the same time, the QRS complex is a feature that starts from the Q point to the end of the S wave with a duration between 80–120 milliseconds (ms) [54]. TRecording2 ECG signals take four electrodes placed at specific points on the body. Each configuration of the electrode location will represent a different ECG signal, commonly referred to as a lead. Twelve leads can be recorded at one time by the Electrocardiograph. However, this study will use two leads, namely MLII and V5. The MLII signal will then be used as the lead one signal, and the V5 signal will be used as the lead two signal.

- MLII: obtained by placing electrodes on the patient’s chest
- V1: obtained by placing electrodes on the intercostal space (ICS), and the right side of the sternum on the ribs

**Fig 4:**
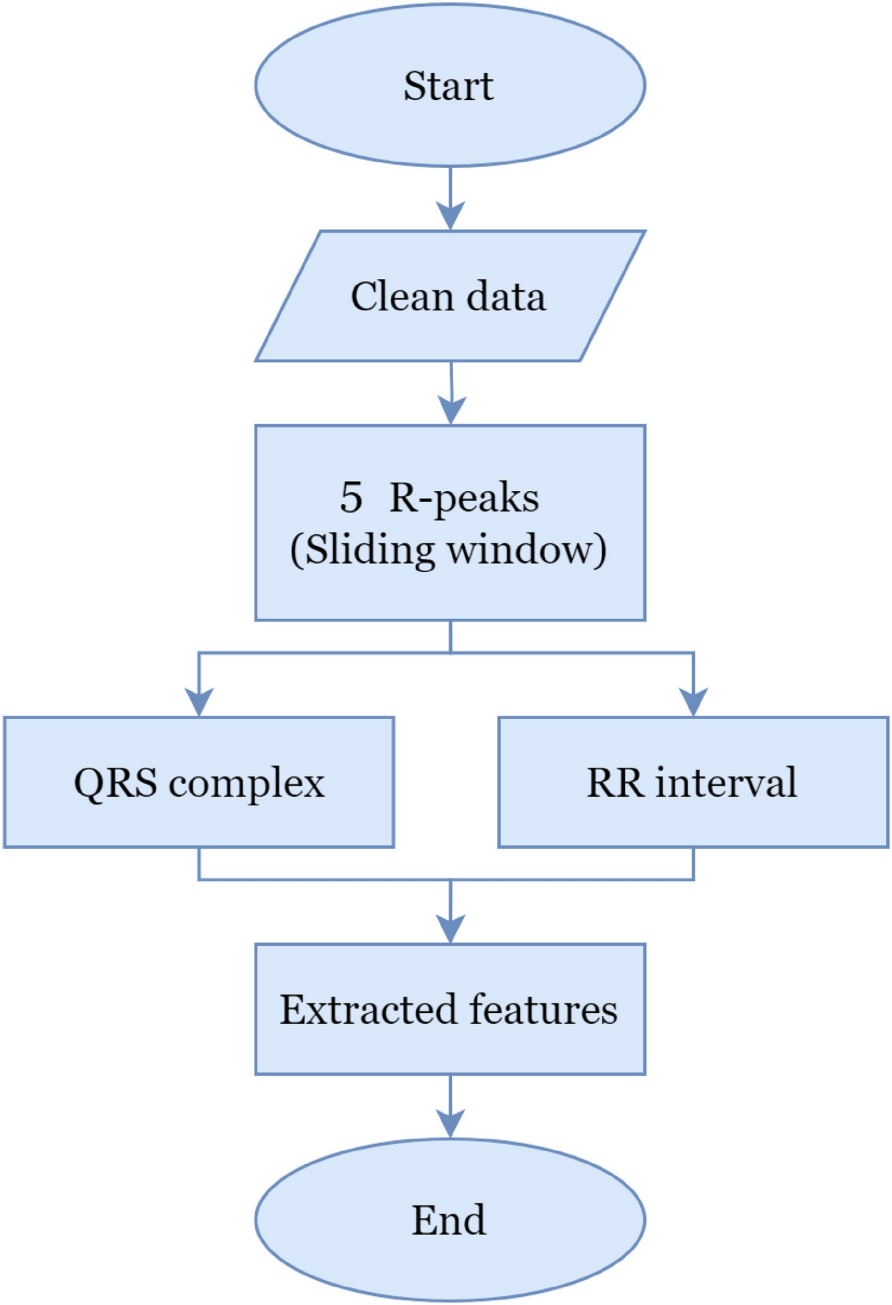
Proposed feature extraction technique.

**Fig 5:**
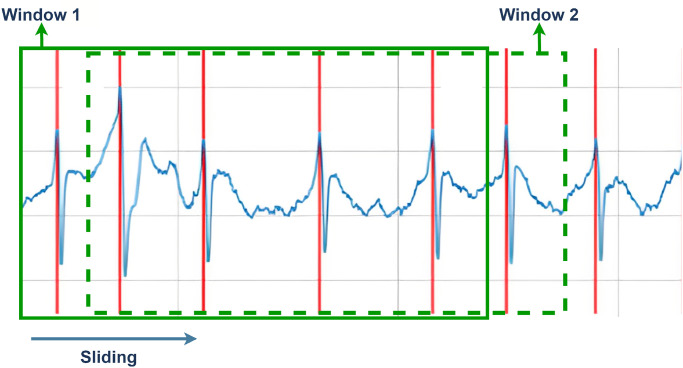
Sliding window technique process.

In the feature extraction stage, this research proposes a new technique, as shown in Fig 4. In extracting QRS complex and RR interval features, this research proposes applying a sliding window technique to each of the 5 R peaks. According to [55] sliding window is a technique used in digital signal processing to analyze signals in a specific time frame. The sliding window representation of a signal is a signal description in time and frequency simultaneously, and it is complete in the sense that the signal can be reconstructed from its sliding-window spectrum. This technique is applicable in specific scenarios where the size of the window for computation is fixed throughout the complete nested loop. Fig 5 explains the work flow of the sliding window technique. Furthermore, based on [56] Algorithm 1 is a sliding window pseudocode, which has also been implemented in various scenarios according to guidance from [57].

**Algorithm 1**: Sliding window pseudocode

Input array *A* of length *n*, window size *k*

$window\_sum=\sum_{i=0}^{k-1}A\left[i\right]$

*max*_*sum* = *window*_*sum*

**for** *i* ← 0 to *n* − *k* **do**

 *window*_*sum* = *window*_*sum* − *A*[*i*] + *A*[*i* + *k*]

 *max*_*sum* = max(*max*_*sum*, *window*_*sum*)

**end for**

**return** *max*_*sum*

### Section 4.3: Classification

#### Section 4.3.1: Data distribution visualization

Visualization of the data distribution carries out at this stage. The visualization results were then analyzed to determine the appropriate classification technique for this study.

#### Section 4.3.2: Data split

In this process, a data split process is carried out with a ratio of 70:30, meaning that training data uses 70% of the dataset, and test data uses 30%. This process is carried out so that the experimental results avoid overfitting.

#### Section 4.3.3: Ensemble method

**Fig 6:**
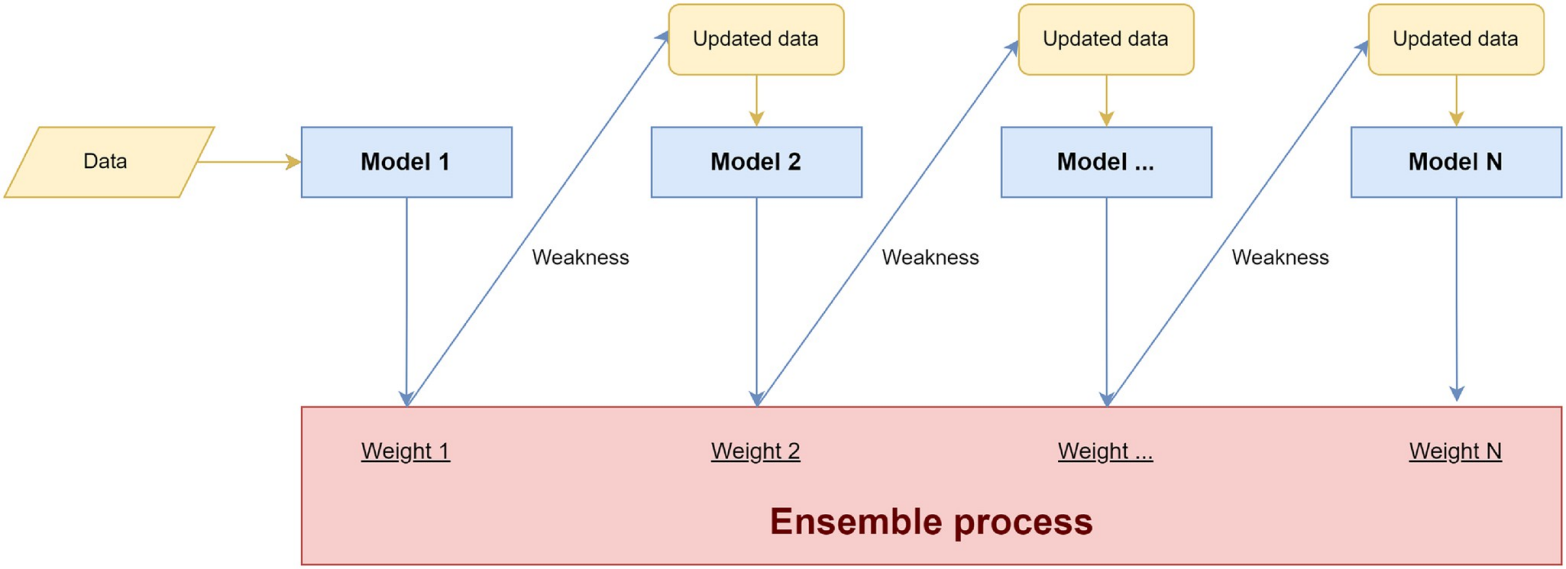
Boosting.

In this study, the ensemble learning technique used is Boosting. Boosting is a technique of ensemble learning which is non-independent and homogenous. Non-independent means that the algorithm can learn from mistakes made by previous algorithms. Based on that property, boosting works sequentially then learns the previous algorithm and tries to predict based on the error. The learning process from the previous algorithm aims to get better results. The flow of the Boosting method can be seen in Fig 6.

**Algorithm 2**: Boosting Pseudocode

Initialize weights $W:W_{1},W_{2},...,W_{n}=\frac{1}{n}$

**for** *i* in [1, *M*] **do**

{M is model classifiers}

*fit weak classifier C*^*i*^ *with sample weights W*

$Error^{i}=\frac{\Sigma_{j=1}^{n}W_{j}^{1}\left(C^{i}\left(x_{j}\right)\neq Y_{j}\right)}{\Sigma_{j=1}^{n}W_{j}}$

$a^{i}=log(\frac{1-Error^{i}}{Error^{i}}+log\left(K-1\right)$ {coefficients for C}

$W_{j}=W_{j}*e^{a^{i}+l(C^{i}\left(x_{j}\right)\neq Y_{j}}$ for *W*_*j*_ ∈ *W* {Update weights}

*W* = *W* − *mean*(*W*) {Normilize weights}

**end for**

*Prediction* : $Y_{j}=max(\Sigma_{i=1}^{i}a_{i}l\left(C^{i}\left(x_{j}\right)=k\right))$

Algorithm 2 is the pseudocode of the boosting algorithm. Where it is explained that the first stage is to initialize the weights on the data, then the data enters it into an iteration consisting of modeling and updating the weights. Each weight will be updated before entering the next model, this weight update uses weaknesses or errors from the previous weights. Finally, these models will be combined with an ensemble to produce predictions [58].

### Section 4.4: Performance matrix

Evaluation metrics used in conducting this research are sensitivity, specificity, and accuracy. These three evaluation metrics were chosen because these metrics have been widely used in research on detection in the health sector. Sensitivity tests the algorithm’s ability to predict arrhythmia signals accurately. Specificity tests the algorithm’s ability to predict standard signals correctly. To perform calculations using the sensitivity, specificity, and accuracy equations, use the Confusion Matrix, which can be seen in Table 1. TP, TN, FN, and FP are the sum of true positive, true negative, false negative, and false positive. This evaluation method has been carried out in several studies [59–62].

**Equation of Accuracy**

$$\begin{array}{c} accuracy=\frac{TP+TN}{TP+FP+FN+TN} \end{array}$$ (1)

**Equation of Specificity**

$$\begin{array}{c} specificity=\frac{TN}{TN+FP} \end{array}$$ (2)

**Equation of Sensitivity**

$$\begin{array}{c} sensitivity=\frac{TP}{TP+FN} \end{array}$$ (3)

**Table 1:** Confusion matrix.

		True diagnosis	
		Positive	Negative
Screening test	Positive	TP	FP
	Negative	FN	TN

## Section 5: Results

### Section 5.1: Pre-Processing

This study uses datasets from AFDB MIT-BIH [46] and MITDB-MIT-BIH [63]. In the MITDB data, this study extracted two types of arrhythmia (PAC and PVC, with symbols’ A’ and’ V’ respectively) and normal data with symbols’ N.’ As for AFDB data, this study extracts AF data with the symbol’ AFIB’ and normal data’ N.’ According to each record’s label, MITDB and AFDB data are read. Table 2 is detailed information about the data used.

**Table 2:** Detailed information on the data used.

Class	Database	Raw Data	Number of Samples	Data Length(s)
AF	AFDB-MITBIH	22 record	484 samples	4840 s
PVC	MITDB-MITBIH	47 record	180 samples	1800 s
PAC	MITDB-MITBIH	47 record	180 samples	1800 s

**Fig 7:**
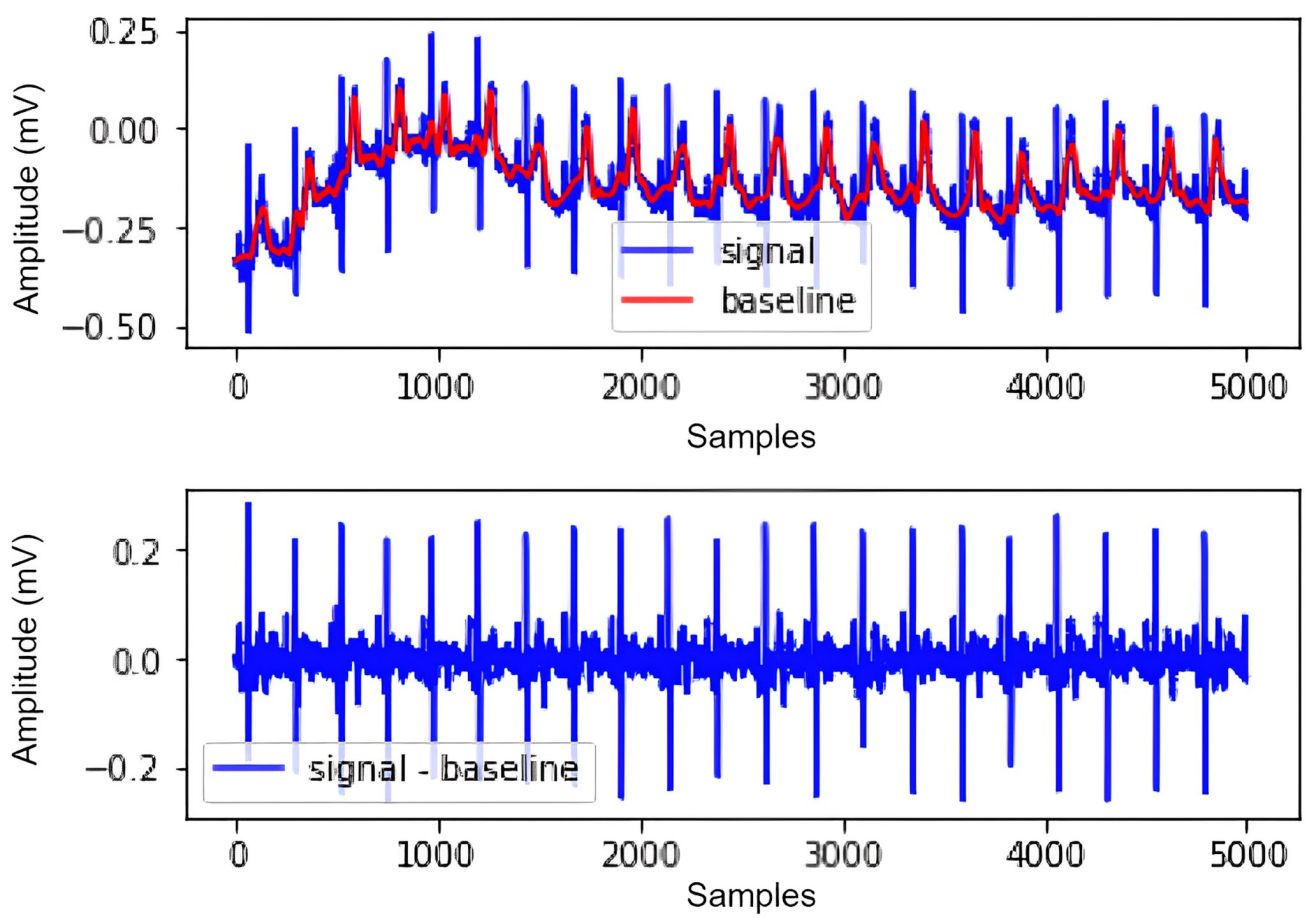
Baseline wander denoising.

Denoising is used to remove noise in AFDB and MITDB signals. The denoising used in this research is the Adaptive Filter method. Besides this, the 5-point derivative [51] is used to remove baseline wander in the signal, making it easier to determine the QRS complex. The first signal in Fig 7 is a signal that has not been denoised. In this signal, there is also baseline wander marked by red. Meanwhile, the second signal is the result of the baseline wander process. As shown in Fig 7, the peak of the R signal is more stable than the first signal.

### Section 5.2: Feature extraction

The Pan-Tompkins algorithm is commonly used to detect R-peaks, QRS complex and extract RR intervals [20]. The algorithm uses the slope, amplitude, and width of the ECG signal [64].

For this reason, this study uses the Pan-Tompkins algorithm to extract two dynamic ECG features, namely RR Interval and QRS complex, on PAC and PVC data. In extracting PAC, PVC, and AF data, the data is processed every 10 seconds of signal length. However, the features used for PAC and PVC are somewhat different from those for AF. PAC and PVC use the long RR interval and QRS complex features, while AF uses the average RR interval and average QRS complex features per 10 seconds of the signal. This is done to see the irregularity of the ECG signal when AF onset occurs [65].

**Fig 8:**
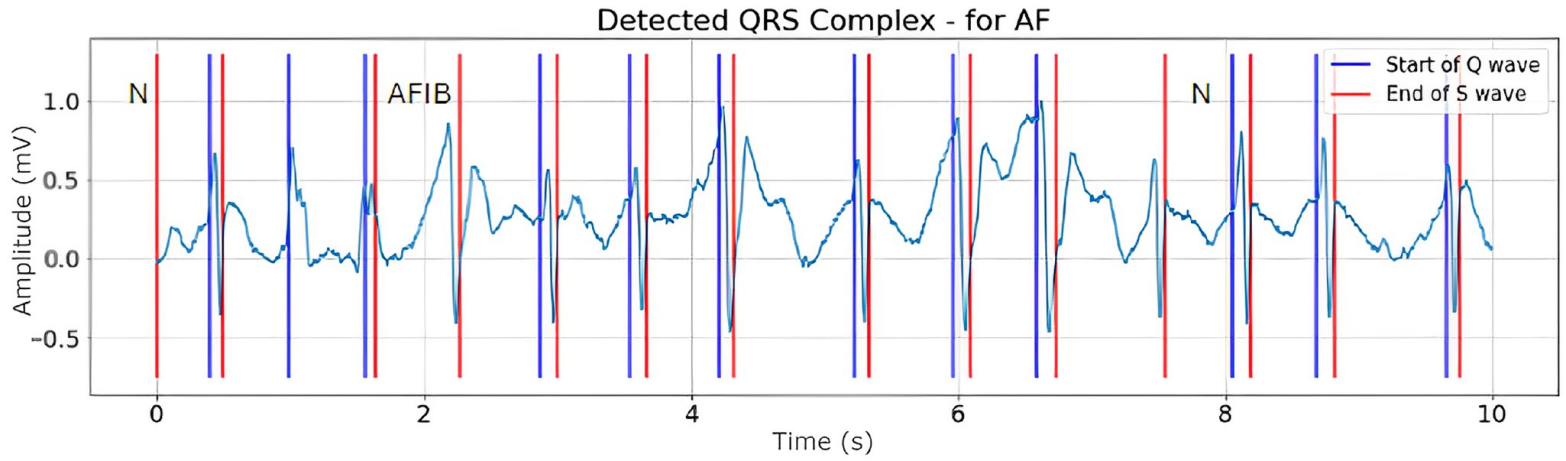
QRS complex detection for AF signal (10 seconds).

**Fig 9:**
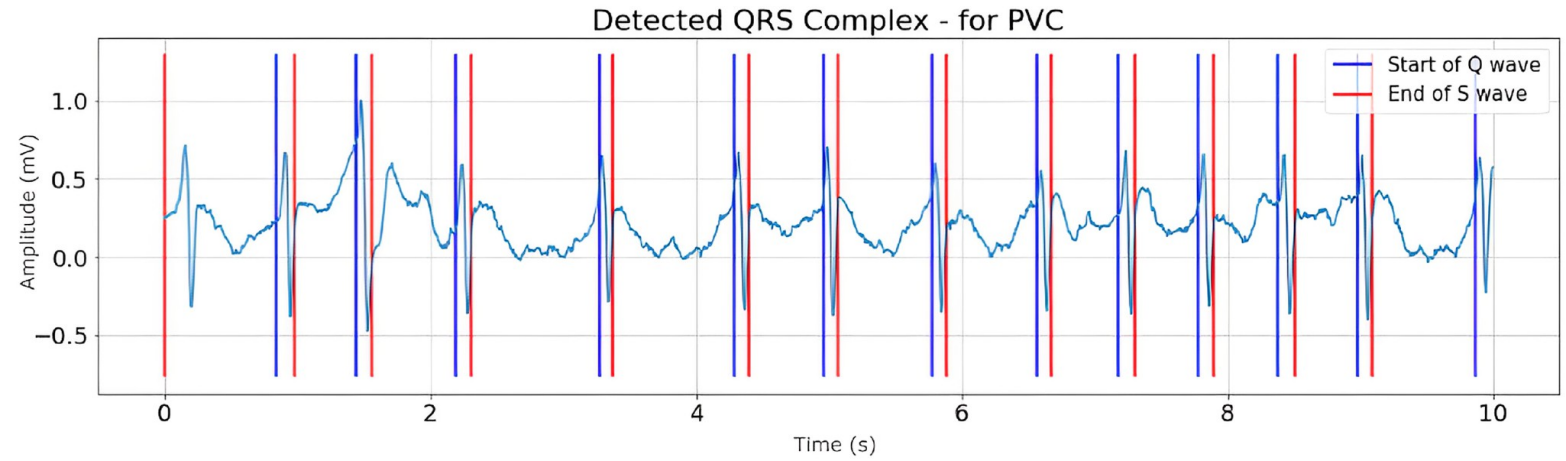
QRS complex detection for PVC signal (10 seconds).

**Fig 10:**
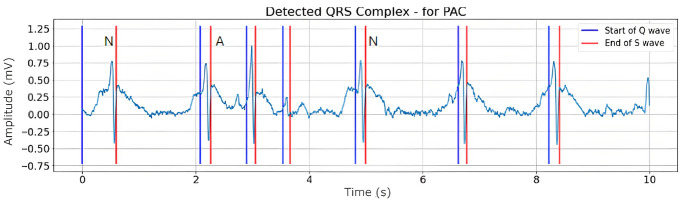
QRS complex detection for PAC signal (10 seconds).

**Fig 11:**
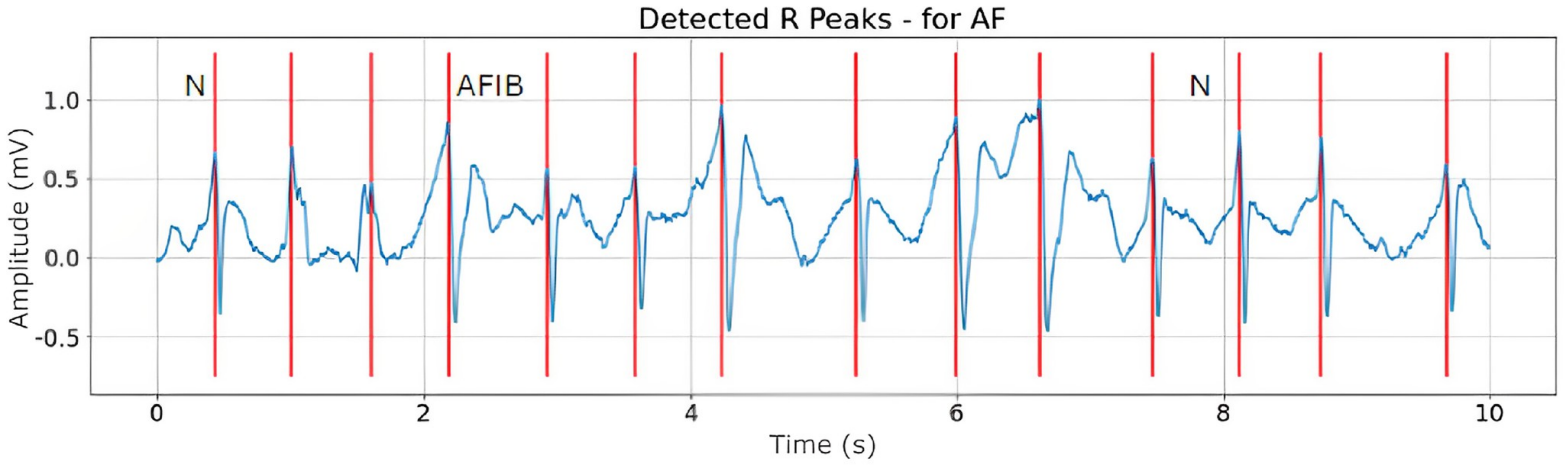
RR interval detection for AF signal (10 seconds).

**Fig 12:**
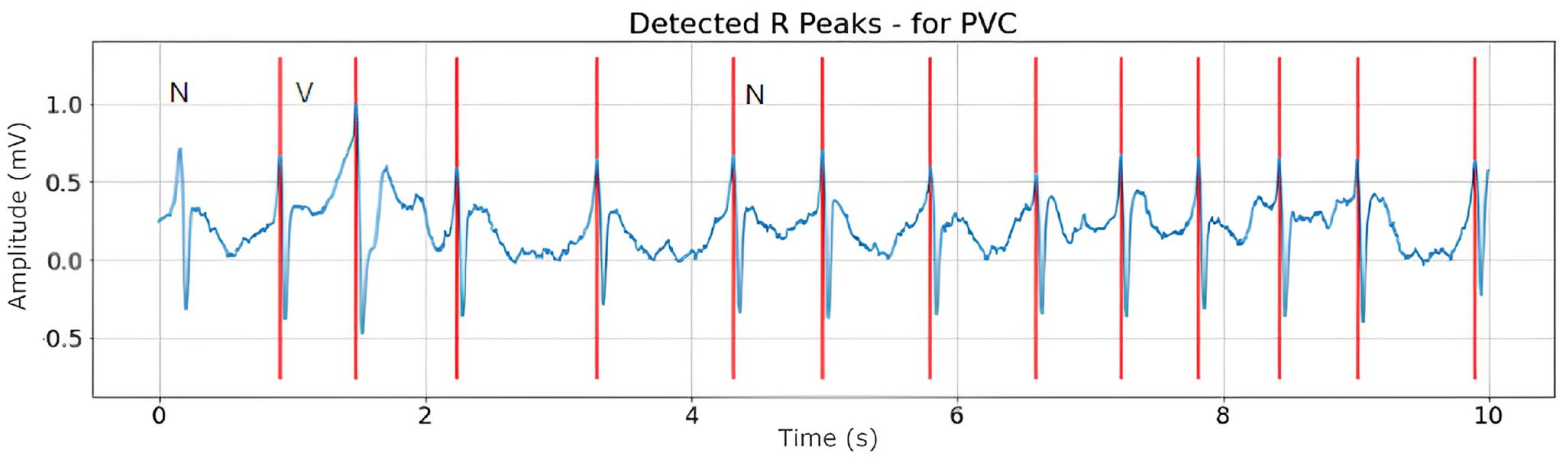
RR interval detection for PVC signal (10 seconds).

**Fig 13:**
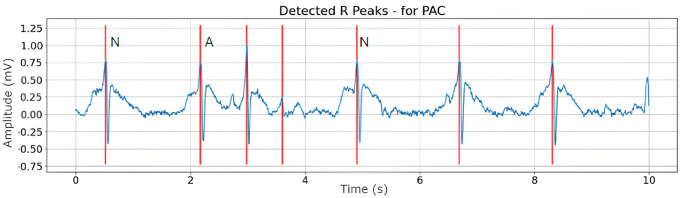
RR interval detection for PAC signal (10 seconds).

Figs 8–10 are illustrations of two-lead ECG signals from three classes of arrhythmia, namely AF, PVC, and PAC, which have been detected by the QRS complex using the Pan-Tompkins algorithm. On the other hand, Figs 11–13 are illustrations of AF, PVC, and PAC signals where the RR interval has been successfully detected. The RR interval is calculated from the distance between the R peak and the following R peak [66].

### Section 5.3: Result of the first scenario

**Fig 14:**
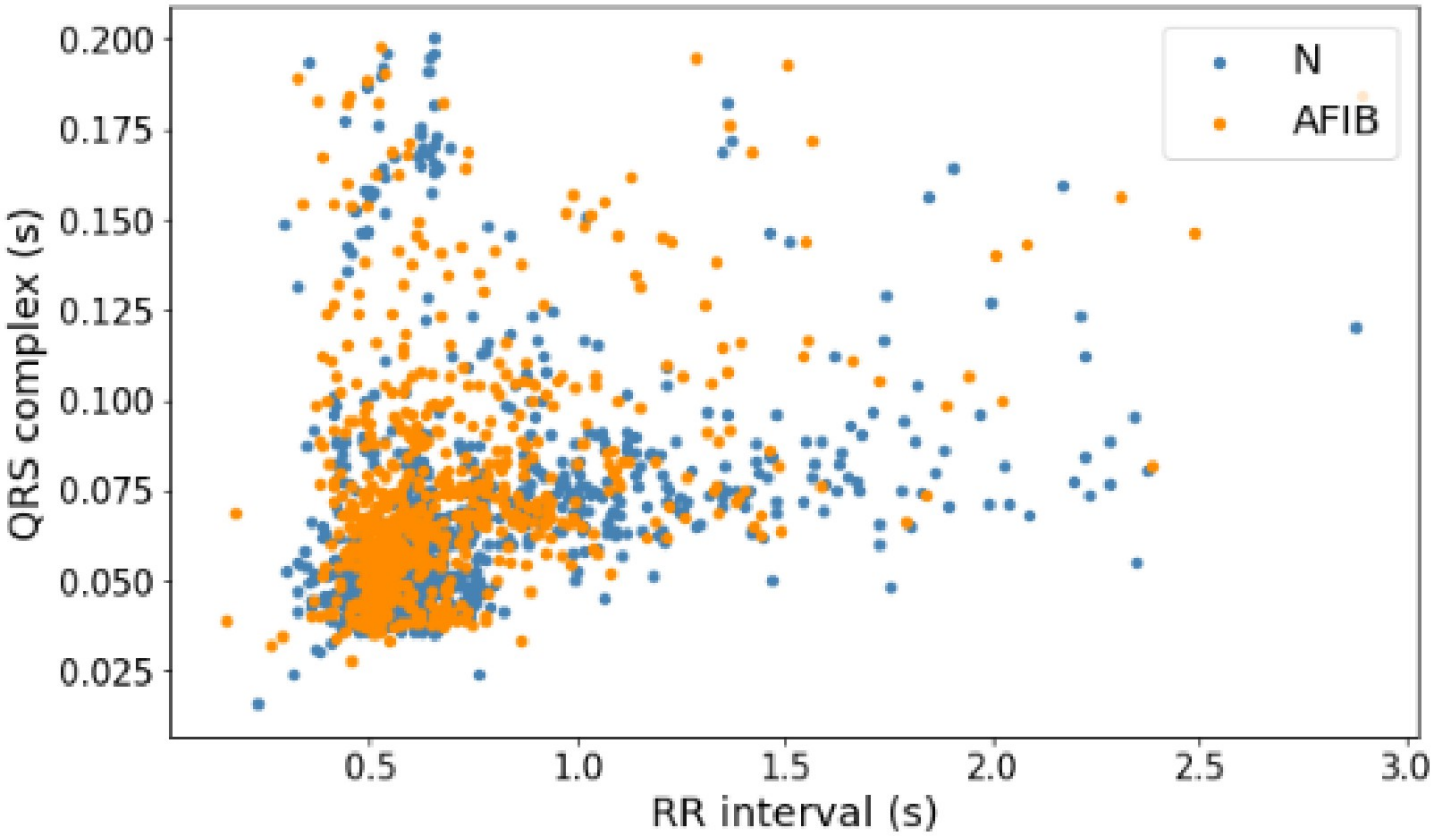
Distribution of PVC data (V) and normal data (N) with RR interval and QRS complex features (record 108).

**Fig 15:**
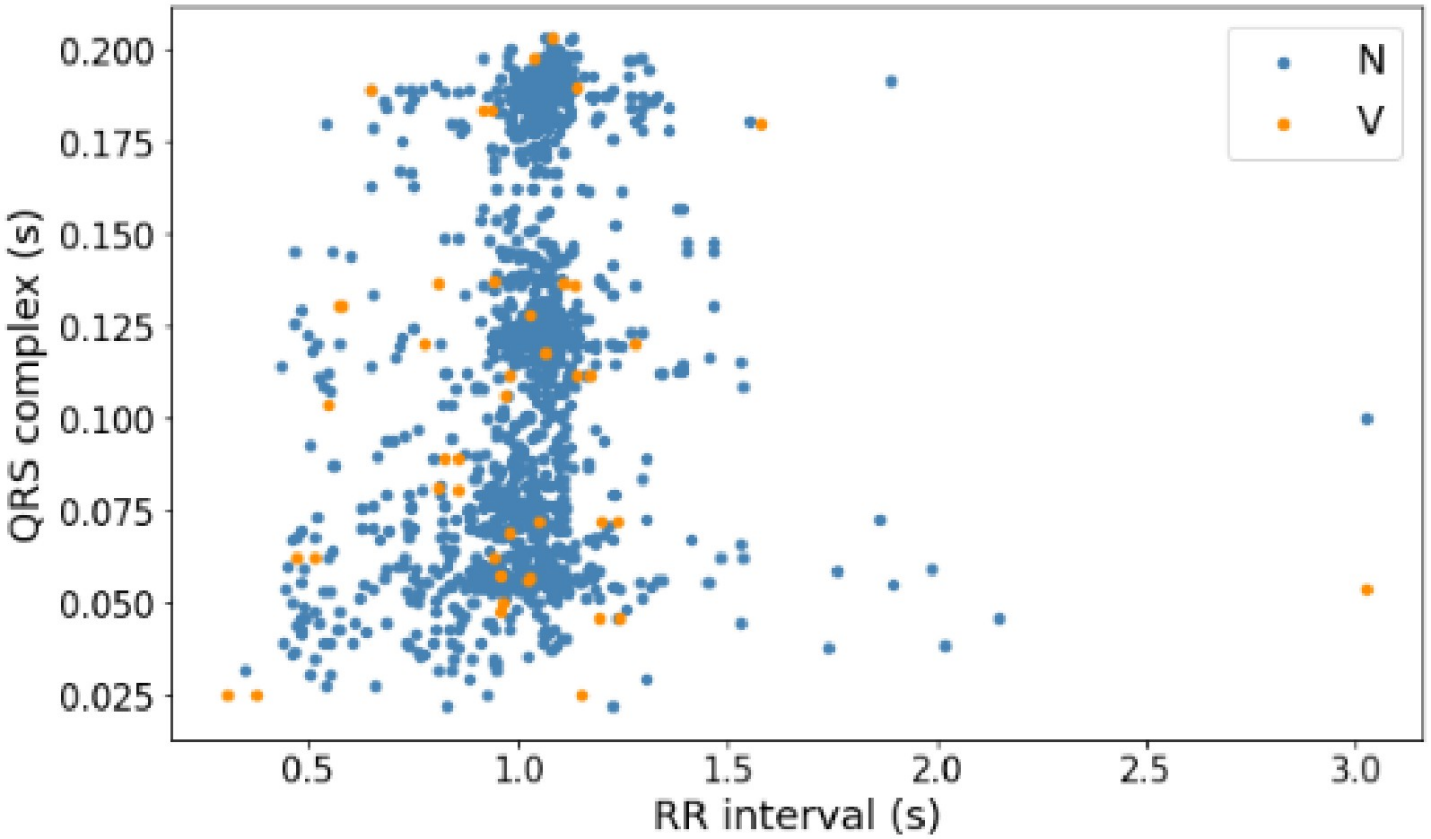
Distribution of PAC data (A) and normal data (N) with RR interval and QRS complex features (record 108).

**Fig 16:**
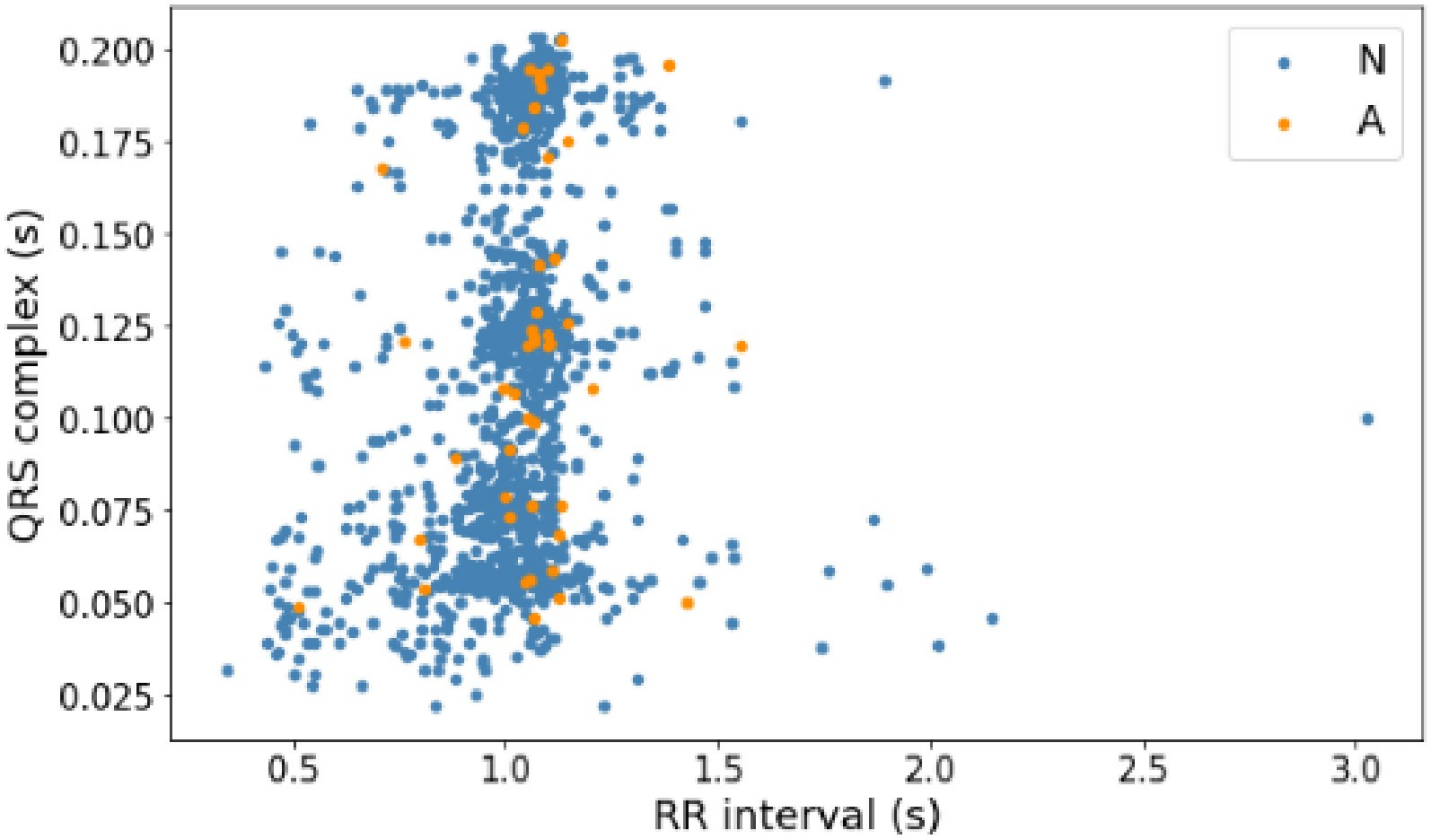
Distribution of AF data (AFIB) and normal data (N) with RR interval and QRS complex features (Records 04043 and 06995).

Before building a model to predict arrhythmia, this research analyzes the distribution of data on each signal based on the RR interval and QRS complex features to choose the suitable algorithm so that it can produce high accuracy. Fig 14 shows the PVC data distribution with normal data on the MITDB record number 108 signal. Then, Fig 15 shows the distribution of PAC data with normal data on the same signal. Last, Fig 16 is the distribution of AF data with normal data on AFDB signal numbers 04043 and 06995. The three figures above (Figs 14–16) show that the AF, PAC, and PVC signals are close. So selecting a suitable classifier algorithm to produce high accuracy takes much work.

The process after looking at the data distribution is to divide the data distribution into 70% training data and 30% test data. Ensemble learning with the boosting model is then used as a classifier algorithm on training data. The boosting model used is the AdaBoost classifier with base_estimator = decision tree. The resulting ensemble learning-based model processes 30% of the test data.

**Fig 17:**
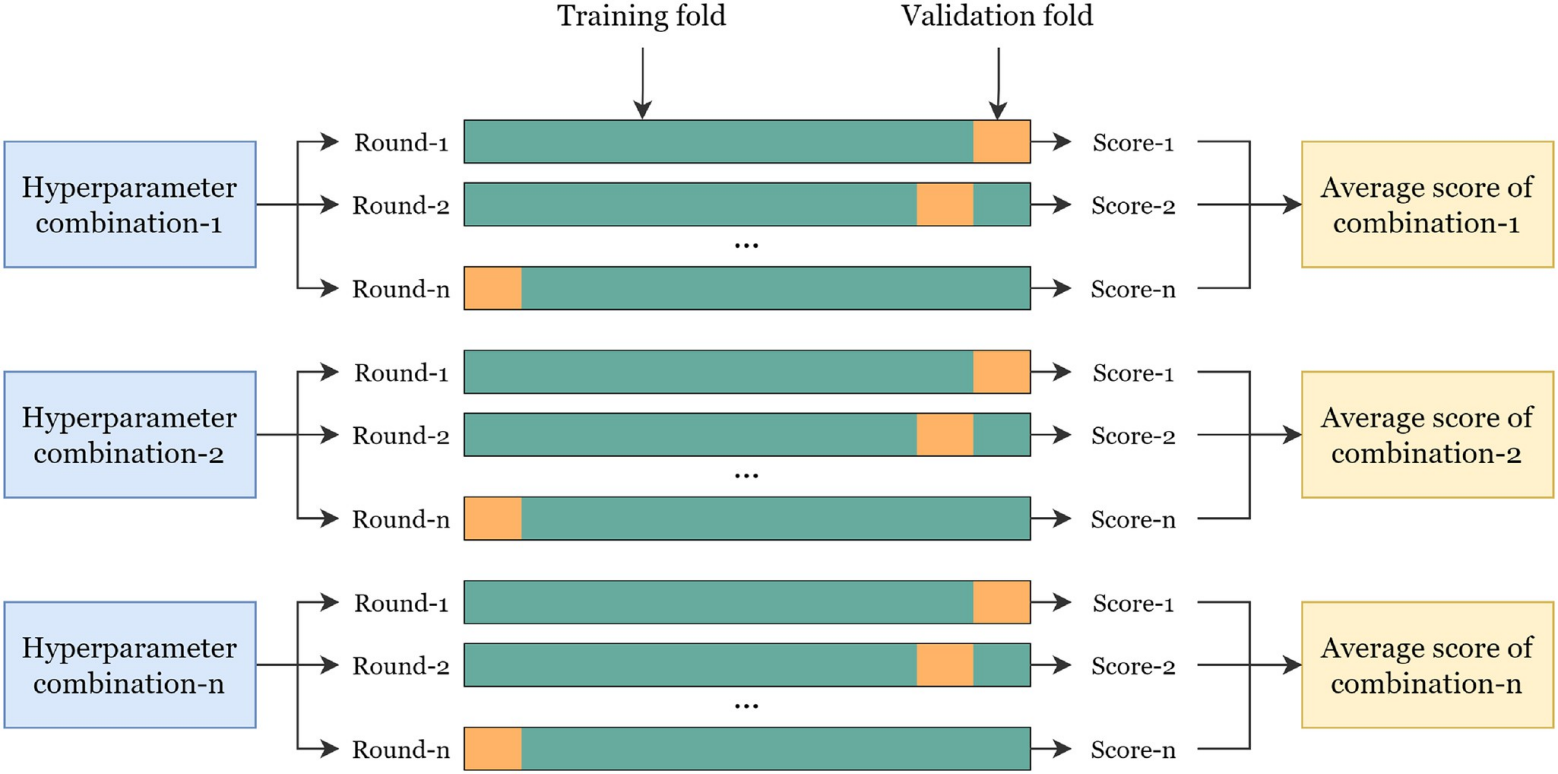
GridSearchCV workflow.

This study also uses hyperparameter tuning to improve the boosting model’s performance in detecting three arrhythmia. GridSearchCV is a tuning method used in this study. This method is also used by [67]. GridSearchCV can be used to find the right combination of values in parameters to produce better accuracy [67]. The GridSearchCV boosting model finds the best value for the base_estimator, n_estimator, and learning_rate parameters. Three classic machine learning models (decision tree, SVM, logistic regression) are the best base_estimator candidates. The results obtained from the best parameter search by GridSearchCV on the Fine-Tuned Boosting (FTBO) model are base_estimator = Decision tree, n_estimator = 10, and learning_rate = 1.0. In addition, cross-validation is also used to separate data into subsets which are then used in the validation process [68]. Fig 17 shows how GridSearchCV and cross-validation work in finding good parameter values.

Tables 3–5 are the results of arrhythmia detection performance (AF, PVC, and PAC) based on the boosting model with hyperparameter: n_estimators = 10, base_estimator = Decision Tree, and learning_rate = 1.0. The algorithm was tested using three evaluation metrics: sensitivity, specificity, and accuracy.

**Table 3:** Boosting model performance without tuning and with tuning to detect AF signals.

Algorithm	Lead	Specificity	Sensitivity	Accuracy
**Boosting model**	**Lead 1**	85%	90%	89%
	**Lead 2**	69%	97%	90%
**FTBO model**	**Lead 1**	98%	**100%**	**99%**
	**Lead 2**	**100%**	98%	98%

**Table 4:** Boosting model performance without tuning and with tuning to detect PVC signals.

Algorithm	Lead	Specificity	Sensitivity	Accuracy
**Boosting model**	**Lead 1**	73%	78%	76%
	**Lead 2**	70%	85%	78%
**FTBO model**	**Lead 1**	**99%**	**99%**	**99%**
	**Lead 2**	99%	99%	99%

**Table 5:** Boosting model performance without tuning and with tuning to detect PAC signals.

Algorithm	Lead	Specificity	Sensitivity	Accuracy
**Boosting model**	**Lead 1**	56%	82%	70%
	**Lead 2**	57%	**80%**	70%
**FTBO model**	**Lead 1**	**96%**	75%	85%
	**Lead 2**	95%	76%	**85%**

**Fig 18:**
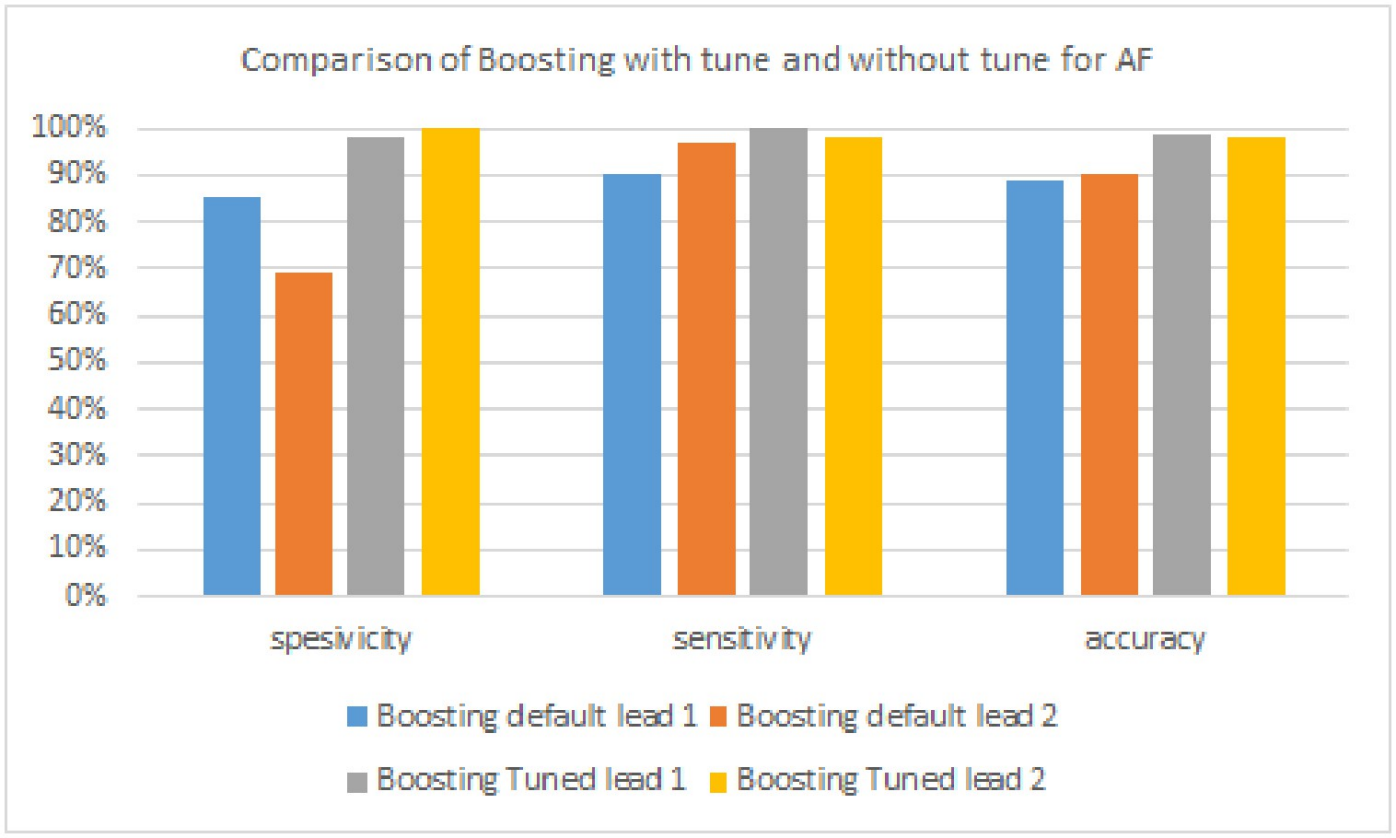
Comparison of boosting with tuning and without tuning for AF signals.

Table 3 and Fig 18 are the results of AF signal detection. The algorithm with the highest sensitivity is Boosting with a tuning of 100% in the lead 1. On the other hand, the algorithm that has the highest specificity is Boosting, with a tuning of 100% in lead 2. Next, the algorithm with the highest accuracy is Boosting, with a tuning of 99% in leads 1.

**Fig 19:**
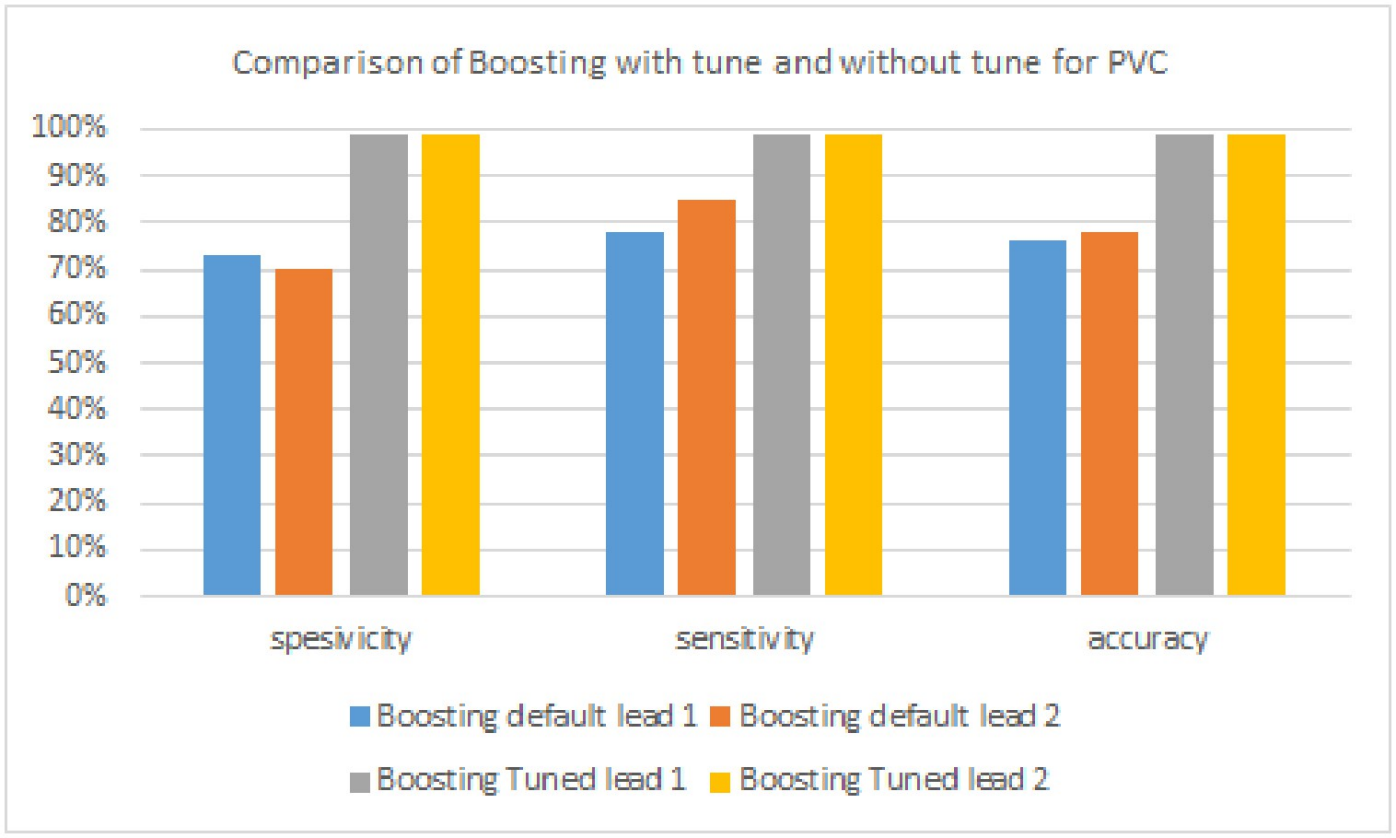
Comparison of boosting with tuning and without tuning for PVC signals.

Table 4 and Fig 19 are the results of PVC signal detection. The algorithm with the highest sensitivity is Boosting, with a tuning of 99% in lead 1. The algorithm with the highest specificity is Boosting, with a tuning of 99% in lead 1. Then, the algorithm with the highest accuracy is Boosting with a tuning of 99% on lead 1.

**Fig 20:**
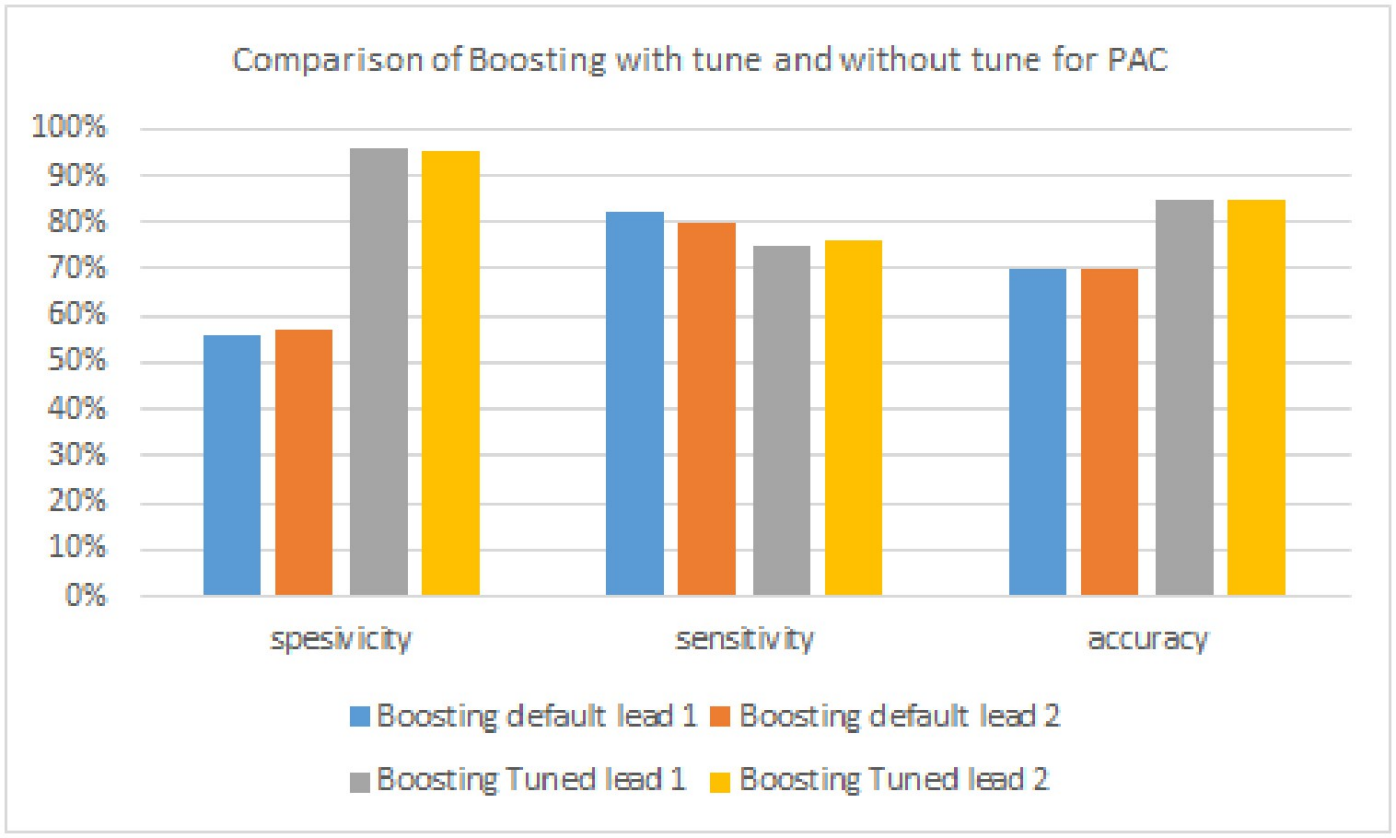
Comparison of boosting with tuning and without tuning for PAC signals.

Table 5 and Fig 20 are the results of PAC signal detection. The algorithm with the highest sensitivity is Boosting without tuning of 80% in lead 2. The algorithm with the highest specificity is Boosting, with a tuning of 96% in lead 1. Next, the algorithm with the highest accuracy is boosted with tuning in lead 2 of 85%. Based on the results of the highest evaluation metric, the highest scores were generated from the boosting model with tuning compared to without tuning. However, for PAC signals, the highest sensitivity results are produced by the boosting model without tuning.

### Section 5.4: Result of the second scenario

**Fig 21:**
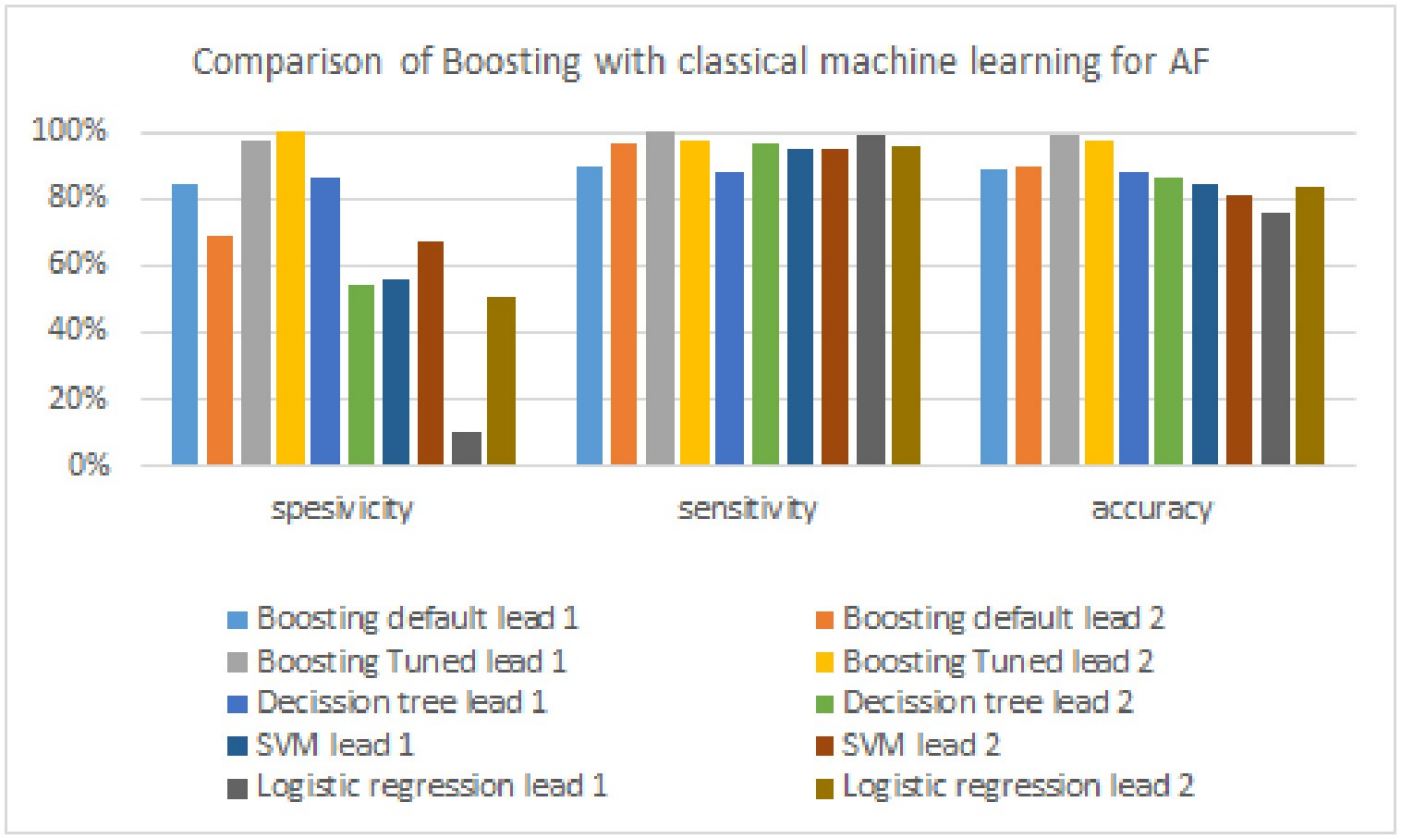
Comparison of boosting with classical machine learning models for AF.

**Fig 22:**
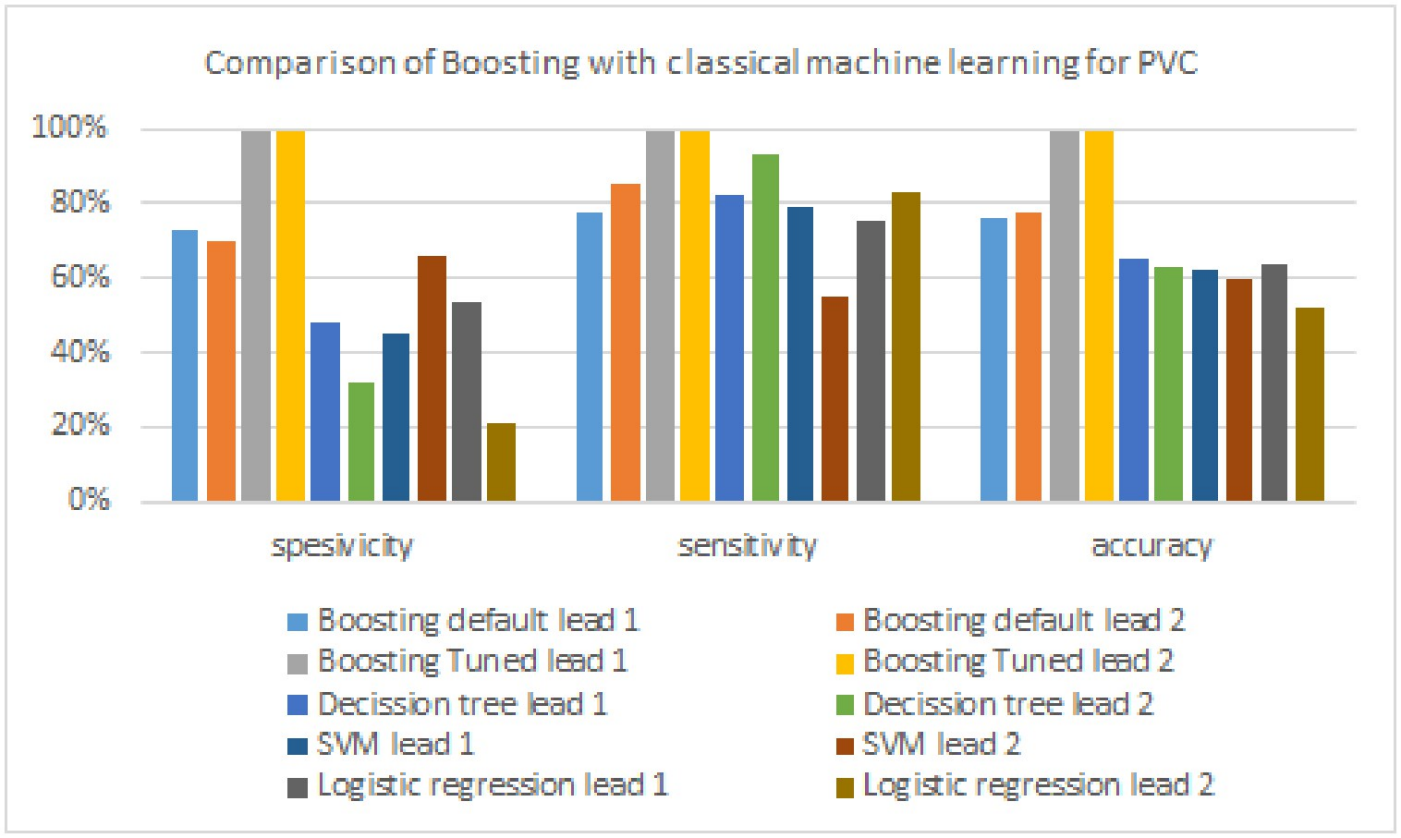
Comparison of boosting with classical machine learning models for PVC.

**Fig 23:**
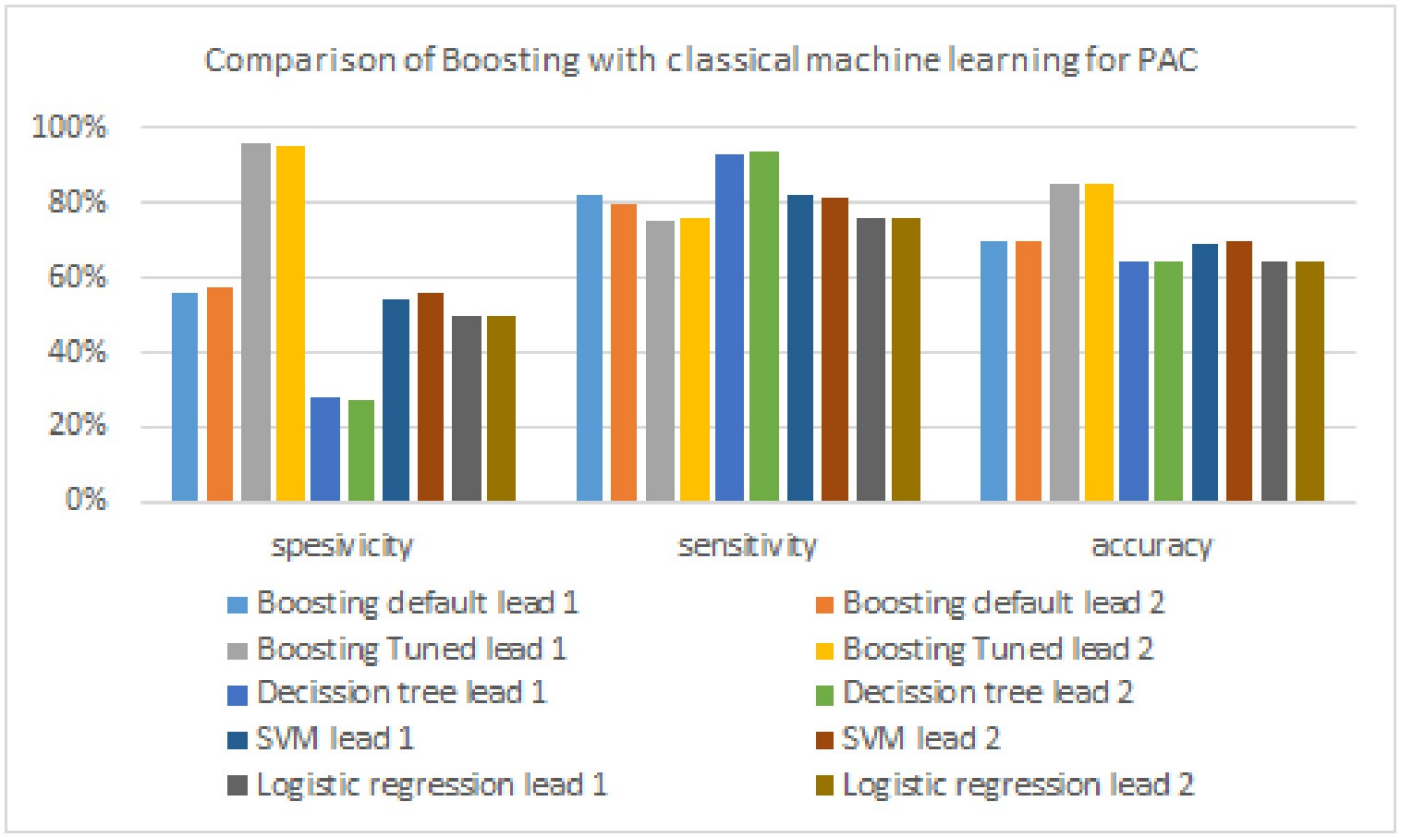
Comparison of boosting with classical machine learning models for PAC.

This research performs a comparative analysis of the results of arrhythmia detection between the boosting model, the FTBO model, and the classical machine learning algorithm used as a candidate base_estimator in the FTBO model, namely decision tree, SVM, and logistic regression. Tables 6 to 8 and Figs 21 to 23 illustrate the performance of the boosting model compared to classic machine learning.

**Table 6:** The performance of boosting models and classical machine learning models for detecting AF signals.

Algorithm	Lead	Specificity	Sensitivity	Accuracy
**Boosting model**	**Lead 1**	85%	90%	89%
	**Lead 2**	69%	97%	90%
**FTBO model**	**Lead 1**	98%	**100%**	**99%**
	**Lead 2**	**100%**	98%	98%
**Decision Tree**	**Lead 1**	86%	88%	88%
	**Lead 2**	54%	97%	86%
**SVM**	**Lead 1**	56%	95%	85%
	**Lead 2**	67%	95%	81%
**Logistic Regression**	**Lead 1**	10%	99%	76%
	**Lead 2**	51%	96%	84%

**Table 7:** The performance of boosting models and classical machine learning models for detecting PVC signals.

Algorithm	Lead	Specificity	Sensitivity	Accuracy
**Boosting model**	**Lead 1**	73%	78%	76%
	**Lead 2**	70%	85%	78%
**FTBO model**	**Lead 1**	**99%**	**99%**	**99%**
	**Lead 2**	99%	99%	99%
**Decision Tree**	**Lead 1**	48%	82%	65%
	**Lead 2**	32%	93%	63%
**SVM**	**Lead 1**	45%	79%	62%
	**Lead 2**	66%	55%	60%
**Logistic Regression**	**Lead 1**	54%	75%	64%
	**Lead 2**	21%	83%	52%

**Table 8:** The performance of boosting models and classical machine learning models for detecting PAC signals.

Algorithm	Lead	Specificity	Sensitivity	Accuracy
**Boosting model**	**Lead 1**	56%	82%	70%
	**Lead 2**	57%	80%	70%
**FTBO model**	**Lead 1**	**96%**	75%	85%
	**Lead 2**	95%	76%	**85%**
**Decision Tree**	**Lead 1**	28%	93%	64%
	**Lead 2**	27%	**94**%	64%
**SVM**	**Lead 1**	54%	82%	69%
	**Lead 2**	56%	81%	70%
**Logistic Regression**	**Lead 1**	50%	76%	64%
	**Lead 2**	50%	76%	64%

As shown in Table 6, the boosting model excels in all evaluation metrics (specificity, sensitivity, and accuracy) compared to classical machine learning algorithms in detecting AF. The classic machine learning algorithm has the highest specificity of 86%, which is 14% lower than boosting. The sensitivity of the boosting model is also 1% superior to the classical machine learning algorithm with the highest AF detection performance (i.e., logistic regression, 99%). Finally, the accuracy metric of the boosting model is 11% superior to the highest classical machine learning algorithm (decision tree, 88%). Fig 21 provides a more precise visual illustration of how the performance of the boosting algorithm compares with classical machine learning in detecting AF.

Table 7 shows the performance results of classic machine learning and boosting models in detecting PVC signals. For specificity, the boosting model outperformed classical machine learning (SVM, 66%) by a difference of 33%. The boosting model also excels in sensitivity of 6%, where the highest sensitivity in classical machine learning is 93%, generated by the decision tree. Then, the boosting model obtains the highest accuracy, which outperforms by 34% compared to classical machine learning (decision tree, 65%). Fig 22 compares the performance of the boosting model with classical machine learning more clearly in the form of a chart.

The comparison of PAC detection performance with the classic boosting and machine learning models can be seen in Table 8 and Fig 23. Compared to classic machine learning, the performance of the boosting model outperformed the two evaluation metrics (specificity and accuracy). The classic machine learning algorithm has the highest specificity, which is 56% which is 40% lower than the boosting model. On the other hand, the algorithm that produces the highest sensitivity is classical machine learning of 94% generated by the decision tree, where the algorithm is 14% superior to the boosting model. Finally, the boosting model again generates the highest accuracy, which outperforms the highest accuracy of classical machine learning (SVM, 70%) by a difference of 15%.

### Section 5.5: Result of the third scenario

The boosting and FTBO models were also compared with the other ensemble models, namely bagging and stacking. The bagging model uses the bagging classifier, and the stacking uses the stacking classifier. To get a fair comparison, the bagging and stacking models are also tuned using GridSearchCV. Based on the results of GridSearchCV, the Fine-Tuned Bagging (FTBA) model uses hyperparameters with a value of n_estimators = 10, base_estimator = Decision Tree, and max_samples = 10. Meanwhile, the Fine-Tuned Stacking (FTST) model uses hyperparameters with final_estimator = Decision Tree and stack_method = predict_proba.

**Fig 24:**
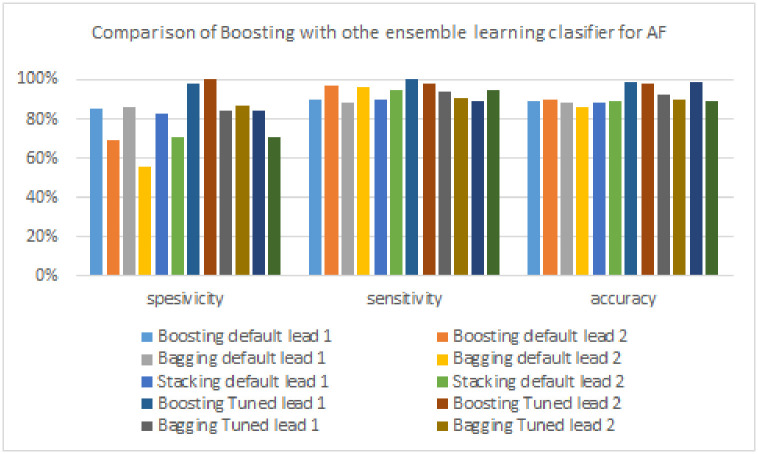
Comparison of Boosting with other ensemble learning clasifier for AF Signals.

Table 9 summarizes the performance comparison of the boosting model and other ensemble learning-based classification models (bagging and stacking) in detecting AF. The FTBA model yielded a specificity of 87%, which is 13% lower than the FTBO model. For sensitivity, the FTBO model also outperforms the bagging model by a difference of 4%. Then the FTBA model accuracy metric produces the highest accuracy of 92%, 7% lower than the FTBO model. Furthermore, Fig 24 provides a visual illustration of the performance comparison of the boosting model with other ensemble algorithms.

**Table 9:** The performance of boosting models and other ensemble learning models for detecting AF signals.

Algorithm	Lead	Specificity	Sensitivity	Accuracy
**Boosting model**	**Lead 1**	85%	90%	89%
	**Lead 2**	69%	97%	90%
**Bagging model**	**Lead 1**	86%	88%	88%
	**Lead 2**	56%	96%	86%
**Stacking model**	**Lead 1**	83%	90%	88%
	**Lead 2**	71%	95%	89%
**FTBO model**	**Lead 1**	98%	**100%**	**99%**
	**Lead 2**	**100%**	98%	98%
**FTBA model**	**Lead 1**	84%	94%	92%
	**Lead 2**	87%	91%	90%
**FTST model**	**Lead 1**	**84%**	89%	88%
	**Lead 2**	71%	95%	89%

**Fig 25:**
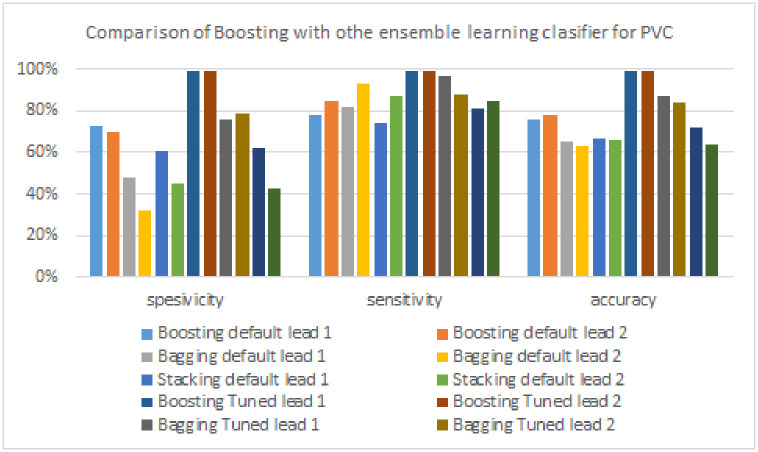
Comparison of Boosting with other ensemble learning clasifier for PVC Signals.

As shown in Table 10, the boosting model excels in all three test metrics (specificity, sensitivity, and accuracy) compared to other ensemble algorithms (bagging and stacking) in detecting PVC signals. The FTBA model yielded the highest specificity of 79%, which is 20% lower than the boosting model. The sensitivity produced by the FTBA model is 97%, which is 2% lower than the boosting model. Furthermore, the highest accuracy was produced by the boosting model with a difference of 12% when compared to the results of other ensemble models (FTBA model, 87%). Fig 25 provides a more precise illustration of the performance comparison of the boosting model with other ensemble models.

**Table 10:** The performance of boosting models and other ensemble learning models for detecting PVC signal.

Algorithm	Lead	Specificity	Sensitivity	Accuracy
**Boosting model**	**Lead 1**	73%	78%	76%
	**Lead 2**	70%	85%	78%
**Bagging model**	**Lead 1**	48%	82%	65%
	**Lead 2**	32%	93%	63%
**Stacking model**	**Lead 1**	61%	74%	67%
	**Lead 2**	45%	87%	66%
**FTBO model**	**Lead 1**	**99%**	**99%**	**99%**
	**Lead 2**	99%	99%	99%
**FTBA model**	**Lead 1**	76%	97%	87%
	**Lead 2**	79%	88%	84%
**FTST model**	**Lead 1**	62%	81%	72%
	**Lead 2**	43%	85%	64%

**Fig 26:**
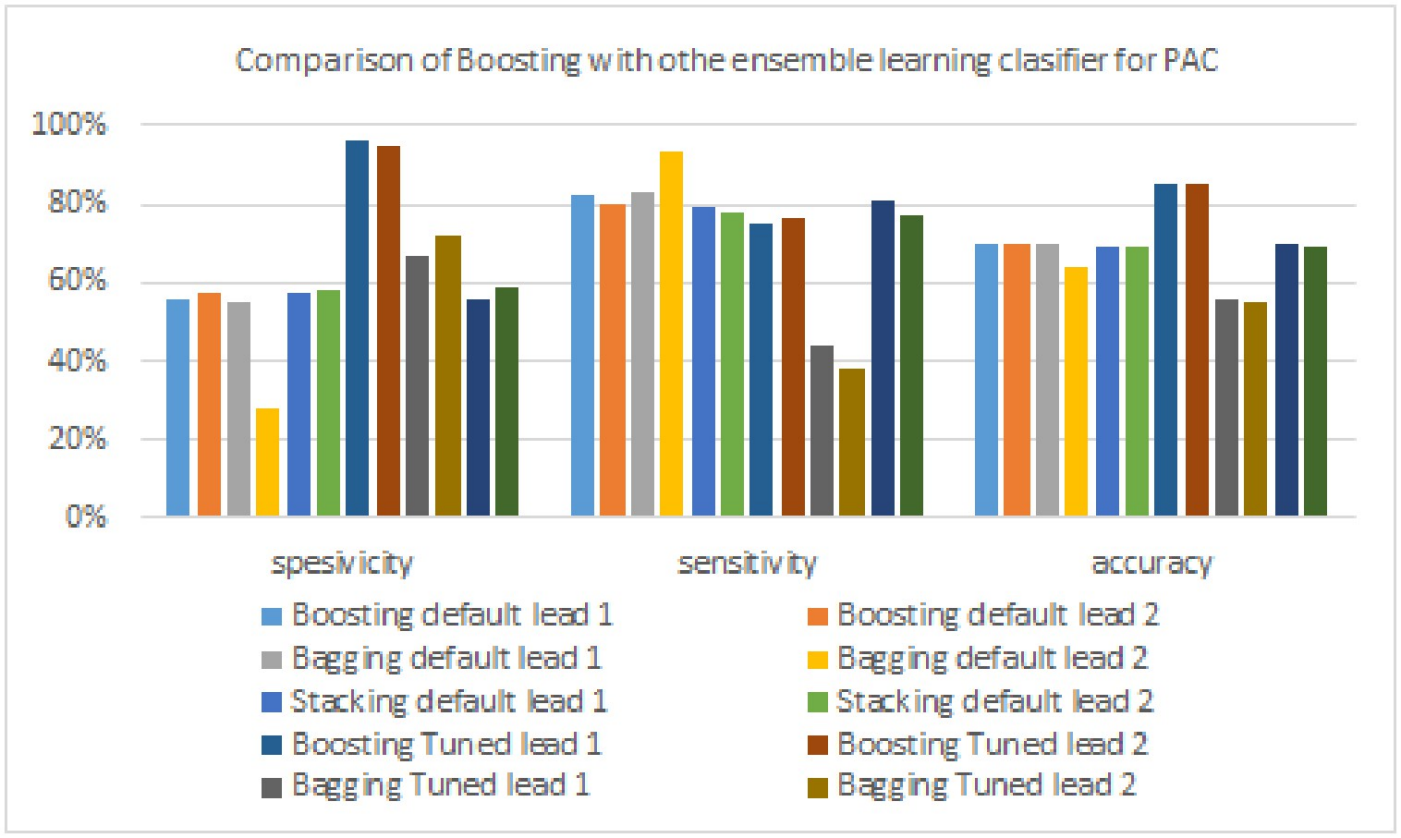
Comparison of Boosting with other ensemble learning clasifier for PAC Signals.

A comparison of the performance of the boosting model with other ensemble models in detecting PAC signals can be seen in Table 11, and a more detailed illustration can be seen in Fig 26. The FTBO model excels in specificity by 24% compared to other ensemble models (FTBA model, 72%). In contrast, the model that produces the highest sensitivity is another ensemble model of 93% produced by the Bagging model, with a difference of 11% compared to the Boosting model. Finally, the highest accuracy is produced by the FTBO model, which is 15% superior to other ensemble models (Bagging model, 70% and FTST model, 70%).

**Table 11:** The performance of boosting models and other ensemble learning models for detecting PAC signals.

Algorithm	Lead	Specificity	Sensitivity	Accuracy
**Boosting model**	**Lead 1**	56%	82%	70%
	**Lead 2**	57%	80%	70%
**Bagging model**	**Lead 1**	55%	83%	70%
	**Lead 2**	28%	**93%**	64%
**Stacking model**	**Lead 1**	57%	79%	69%
	**Lead 2**	58%	78%	69%
**FTBO model**	**Lead 1**	**96%**	75%	85%
	**Lead 2**	95%	76%	**85%**
**FTBA model**	**Lead 1**	67%	44%	56%
	**Lead 2**	72%	38%	55%
**FTST model**	**Lead 1**	56%	81%	70%
	**Lead 2**	59%	77%	69%

### Section 5.6: Performance of boosting compared to state-of-the-art of boosting

#### Section 5.6.1: Comparison of the proposed boosting with other works in detecting arrhythmia using RR interval and QRS complex

In this section, compare the results of our study with several other studies that used the RR interval and QRS complex features in detecting arrhythmia. The boosting model with tuning is used as a comparison because this algorithm is higher than the boosting model without tuning. Table 12 is the result of the comparison of our study with other studies.

**Table 12:** Comparison of the results of this study with other studies based on the feature extraction.

Work	Dataset	Class	Classifier	Spesificity	Sensitivity	Accuracy
Rezaei et.al [41]	UK Biobank	AF	XGBoost	90.9%	98.6%	99%
Plesinger et.al [39]	Physionet/CinC	AF	Bagged tree	77%	86%	-
	Physionet/CinC	Other Arrhythmia	Bagged tree	73%	77%	-
Shi et.al [40]	MIT-BIH	PVC	KNN, SVM, DT, RF	76%	88.1%	74.5%
	MIT-BIH	PAC	KNN, SVM, DT, RF	54.6%	81.8%	
Li et.al [69]	Physionet/CinC	AF	Dilated Residual Neural Network	-	-	84.3%
	MIT-BIH	AF	Dilated Residual Neural Network	-	-	98.4%
Proposed Method	MIT-BIH	AF	FTBO model	100%	100%	99%
	MIT-BIH	PVC	FTBO model	99%	99%	99%
	MIT-BIH	PAC	FTBO model	96%	76%	85%

As shown in Table 12, in the case of AF detection, our proposed boosting model excels in all evaluation metrics (specificity, sensitivity, and accuracy) compared to other studies [39, 41]. The proposed ensemble learning model has 9.1% higher specificity than the XGBoost model used by [41]. The sensitivity of the proposed ensemble learning model also has a higher performance of 1.4% than the model used by [41].

For the case of PVC detection, our proposed ensemble learning model excels in all metrics compared to the model developed by [40]. The specificity of our proposed boosting model is 23% superior. Meanwhile, the superior sensitivity and accuracy are 10.9% and 24.5%, respectively. The proposed ensemble learning model performs better than [40] in the case of PAC detection. Our proposed ensemble learning model is superior to [40] for specificity and accuracy by 41.4% and 10.5%, respectively. Furthermore, this proposed method also outperforms the performance results of Li et al. [69]. Whereas this study is superior by 0.6% on the same dataset, MIT-BIH.

#### Section 5.6.2: Comparison of the proposed boosting with other works in detecting arrhythmia using multiple leads ECG

In this section, compare the results of our study with other studies using multiple leads to ECG signals. A boosting model tuning 2 ECG leads, namely lead one and lead 2, is used in this comparison. Table 13 shows the results of the comparison of our research with other studies such as [12–15, 36, 70].

**Table 13:** Comparison of the results of this study with other studies based on the total leads.

Work	Dataset	Class	Leads	Classifier	Spesificity	Sensitivity	Accuracy
Lee et.al [9]	Chapman University	11 classes	12 leads	XGBoost	-	89.2%	90.46%
Ye et.al [10]	CPSC2018	9 classes	12 leads	XGBoost	-	78.8%	96.4%
Zheng et.al [11]	MIT-BIH	4 classes	12 leads	XGBoost	-	-%	99.2%
Jadhav et.al [14]	UCI	15 classes	12 leads	Random Subspace, PART	-	-	91.11%
Zhou et.al [15]	MIT-BIH	PVC	12 leads	LCNN, LSTM	98.03%	96.42%	98.06%
Manju et.al [36]	UCI	10 classes	12 leads	SMOOST method (random forest)	-	-	93.16%
Mert et.al [12]	MIT-BIH	LBBB, RBBB, AP, PVC, PB	2 leads	Bagging decission tree	99.80%	97.50%	99.51%
Afkhami et.al [13]	MIT-BIH	16 classes	2 leads	Bagging decission tree	-	99.70%	99.70%
Qi et.al [70]	Hercules-3	16 classes	12 leads	9-layer CNN	-	-	98.01%
	Hercules-3	16 class	12 leads	5-layer FCN	-	-	98.67%
Proposed Method	MIT-BIH	AF	2 leads	FTBO model	100%	100%	99%
	MIT-BIH	PVC	2 leads	FTBO model	99%	99%	99%
	MIT-BIH	PAC	2 leads	FTBO model	96%	76%	85%

As shown in Table 13, our proposed ensemble learning model excels across all evaluation metrics compared to other studies in detecting arrhythmia. For specificity, [12, 15] had lower results of 1.97% and 0.2%, respectively. Then the sensitivity metric [15] is 3.58% lower. [12] is 2.5% lower. Furthermore, [13] also has a lower sensitivity of 0.3% than our study. In general, the accuracy of the results of our study is greater than that of other studies. Research by [14] was 8.89% lower. [15] was 1.4% lower. Furthermore, Qi et al. [70] was 0.99% and 0.33% lower. Meanwhile, in [12] and [13], the resulting accuracy is almost identical to the proposed boosting model.

## Section 6: Discussion

This study proposes developing a boosting model to detect three arrhythmia types: AF, PVC, and PAC. Arrhythmia detection was performed on multiple ECG leads (Lead 1 and Lead 2) obtained from the AFDB MIT-BIH dataset for AF signals and MITDB MIT-BIH for PVC and PAC signals. A baseline Wander Filter is used to process the ECG signal in both leads so that the baseline signal is straight. Then a denoising-based adaptive filter is applied to reduce the noise level in the ECG signal in both leads. The Pan-Tompkins algorithm is used to detect fiducial points in the ECG signal. The use of fiducial points can make it easier to extract features based on the RR interval and the width of the QRS complex of the ECG signal. These features are then used as training and testing data for the boosting ensemble classifier. This study also performs tuning on the boosting parameter using the GridSearchCV method. The performance of the boosting model in detecting arrhythmia was then compared with the classic machine learning model and the bagging and stacking model. The experimental results show that the FTBO model performs better in detecting AF-type arrhythmia when compared to the boosting model. The accuracy of the FTBO model is 99% for lead 1 and 98% for lead 2. In detecting PVC signals, the performance of the FTBO model is also better than the boosting model, with an accuracy of 99% for lead one and lead 2. For the PAC case, the performance of The FTBO model also outperforms the boosting model, with an accuracy of 85% for lead one and lead 2.

There are several previous studies similar to this research. Each study produces different evaluation metric values from different models. This study used three previous studies as a reference when compared based on the features used, namely the RR interval and QRS complex. Two of the three studies used the AF signal as a class of detected arrhythmia. First is [41], which proposes a method for classifying arrhythmia, specifically atrial fibrillation (AF) and ventricular arrhythmia signals, with a two-stage ensemble classifier. The ensemble model used is XGBoost, a combination of decision tree algorithms. The research resulted in a Sensitivity of 98.6%, a Specificity of 90.95%, and an Accuracy of 99%. Compared with our study on AF signal detection, our specificity values are superior to 9.1% in sensitivity and 1.4% in specificity, and the same results in accuracy. Second, [39] researched the Holter classification of ECG with arrhythmia class AF data. The algorithms used are parallel CNN and bagged tree ensemble. The proposed bagged tree ensemble model combines simple decision trees, shallow neural networks, and support vector machines with different kernels. The results of the proposed ensemble model are an F1-score of 82%, a sensitivity of 74%, and a specificity of 80%. Our proposed method outperforms sensitivity and specificity by 23% and 14%, compared with our study. In addition, one study used PVC and PAC arrhythmia types as a class of arrhythmia detected. [40] researched inter-patient heart rate classification. This research uses an ensemble learning model based on a combination of KNN, SVM, decision trees, and random forest algorithms. The PVC signal’s specificity is 76%, and the sensitivity is 88.1%. Then, in detecting the PAC signal, the specificity is 54.6%, and the sensitivity is 81.8%. The accuracy of both classes is 74.5%. Proposed method outperformed all three evaluation metrics for the PVC and PAC signals compared to our study. Where, in detecting PVC FTBO model is superior, with 11.9% in sensitivity, 24% in specificity, and 10.5% in accuracy. Whereas in the case of the PAC FTBO, the model excelled at 45.4% in specificity and 10.5% in accuracy. Last, [69] proposed a dilated residual neural network model to detect AF on two datasets. The PhysioNet/Cinc dataset produces an accuracy of 84.3%, while the MIT-BIH produces an accuracy of 98.4%. The proposed method and the FTBO model outperform both results by a difference of 14.3% and 0.6%, respectively.

In addition to comparing based on the features of the ECG signal, this study also compare the performance results of our proposed model based on the number of ECG signal leads. Three studies used 12 lead ECG signals, namely [14, 15, 36]. [14] using a random subspace ensemble classifier to detect 15 classes of arrhythmia. However, this model produces high ROC and AUC values, thus proving that the performance of the subspace random ensemble model requires proper tuning to get the best results. The model produces an accuracy of 91.11%. Compared to the FTBO model’s average accuracy, the study is 3.22% lower. [15] researched PVC detection using an ensemble model that combines LCNN and LSTM. The model produces a sensitivity of 96.43%, a specificity of 98.03%, and an accuracy of 98.06%. The FTBO model excels in all evaluation metrics with a difference of 1.5% in sensitivity, 0.97% in specificity, and 0.94% in accuracy. Then, the [36] study uses the SMOOST method, which applies several algorithms (random forest, decision tree, SVM, and KNN). The highest accuracy produced by the model is 93.16%. Compared to the FTBO model’s average accuracy, the model is 1.17% lower. Additionally, Qi et al. [70] conducted research on AF classification with a proposed ECG database called Hercules-3. This research used two classifiers, namely 9-layer CNN and 5-layer FC Network, producing accuracy of 98.01% and 98.67%, respectively. On the other hand, two studies used two lead ECG signals. First, [12] evaluates the performance of the ensemble method, namely the bagging model, a combination of decision tree algorithms. The study resulted in a sensitivity of 97.50%, a specificity of 99.80%, and an accuracy of 99.51%. The FTBO model excels in two evaluation metrics, specificity, and sensitivity, with a difference of 0.2% and 1.5%, respectively. In comparison, the accuracy of the results obtained is almost the same. Second, [13] also uses an ensemble model, namely the bagging decision tree. The model produces a sensitivity of 99.70% and an accuracy of 99.70%. The FTBO model excels at detecting AF signals by a 0.3% difference.

The proposed boosting model outperforms existing studies primarily due to its adaptive weighting mechanism and effective classification capabilities [71]. Boosting, as a powerful ensemble learning technique, dynamically adjusts the emphasis placed on individual data points during training iterations. This adaptability allows the model to assign higher weights to misclassified instances, enabling it to focus on challenging cases and outliers. The iterative learning process, coupled with the sequential building of weak learners, contributes to a refined and nuanced understanding of the underlying data patterns [72]. Moreover, studies in the field of heart disease detection have consistently demonstrated the efficacy of boosting algorithms [30–33]. These studies have reported high accuracy rates in detecting various cardiac conditions, showcasing the robustness and versatility of boosting in the domain of cardiovascular health. In essence, the boosting model success can be attributed to its adaptability through adaptive weighting and its proven track record of achieving high accuracy across diverse heart disease detection tasks.

This study certainly has several advantages that can be highlighted. Where the proposed ensemble model, namely the FTBO model with RR interval and QRS complex features, can produce optimal performance. In addition, this study also use multiple lead ECG signals (2 leads), where research on arrhythmia detection using multiple lead ECG signals with the application of ensemble learning still needs to be done.

## Section 7: Conclussion and future work

In this study, an improved ensemble learning model, specifically termed the Fine Tuned Boosting (FTBO) ensemble model, is developed for the detection of arrhythmia. The proposed model also uses multiple ECG signal leads, namely MLII and V1. Besides that, this study also use two ECG signal features, namely the RR Interval and QRS complex. This study detected three classes of arrhythmia, namely atrial fibrillation (AF), premature ventricular contractions (PVC), and premature atrial contractions (PAC). To measure FTBO performance, three evaluation metrics, namely specificity, sensitivity, and accuracy are used. This research resulted in the highest specificity value of 100% in lead 2, the highest sensitivity of 100% in the lead 1, and the highest accuracy of 99% in lead 1 to detect AF signals. Then in the case of PVC, it produces the highest specificity of 99% on both leads, the highest sensitivity of 99% on both leads, and the highest accuracy of 99% on both leads. Finally, detecting PAC signals produces the highest specificity of 96% in lead 1, the highest sensitivity of 76% in lead 2, and the highest accuracy of 85% in both leads. When viewed from the value of the test meter obtained, FTBO can be said to have strong performance so that it is accurate enough to detect arrhythmia precisely in the three classes of arrhythmia, namely AF, PVC, and PAC.

In the future, this study can be developed by using a larger amount of data, increasing the number of leads and features of the ECG signal used, and trying to implement this method with several other arrhythmia signals. This study will significantly contribute to arrhythmia research and continue to be developed in the future.

## Supporting information

S1 Dataset Dataset for experiment on the proposed method from MITDB, AFDB, and NSRDB. (PDF)

S1 Graphical abstract (PNG)
