# Supplementary material for: An improved method to detect arrhythmia using ensemble learning-based model in multi lead electrocardiogram (ECG)
Source: PLoS One. 2024 Apr 9;19(4):e0297551. doi: 10.1371/journal.pone.0297551 (PMC11003640; doi:10.1371/journal.pone.0297551)
Supplement: S1 Dataset — (PDF) [file pone.0297551.s001.pdf]

S1 Dataset

The data used in this study is data from the MIT-BIH that include MITDB (Arrhythmia DB), AFDB (Atrial Fibrillation DB) and NSRDB (Normal Sinus Rhythm DB) as in Moody GB and Mark RG [1]. The following is the URL of each database:

1. MITDB is: https://www.physionet.org/content/mitdb/1.0.0/

2. AFDB is: https://physionet.org/content/afdb/1.0.0/

3. NSRDB is: https://physionet.org/content/nsrdb/1.0.0/

The MIT-BIH Arrhythmia Database comprises 48 half-hour, two-channel ambulatory ECG recordings obtained from 47 subjects who were examined by the BIH Arrhythmia Laboratory between 1975 and 1979.

The AFDB is a collection of long-term ECG recordings from 25 subjects with atrial fibrillation (AF), mostly paroxysmal. Each recording spans 10 hours and includes two ECG signals captured at a frequency of 250 Hz, with a 12-bit resolution within the ±10 millivolt range. Each recording encompasses four distinct rhythms: AF (atrial fibrillation), AFL (atrial flutter), Jr (AV junctional rhythm), and N (all other rhythms). However, this study focuses exclusively on the AF rhythm. Notably, two data points, namely numbers 00735 and 03665, are excluded from the analysis as they lack AF samples and have not undergone auditing.

The NSRDB provides 18 long-term ECG records from individuals referred to the Arrhythmia Laboratory at Beth Israel Hospital in Boston (now Beth Israel Deaconess Medical Center). The selected subjects did not exhibit significant arrhythmias and comprised 5 men, aged 26 to 45, and 13 women, aged 20 to 50 years.

Table 1 presents detailed records of the databases utilized in this research.

Table 1. Experimental data for arrhytmia.

NO	ARRHYTMIA DATA RECORD NUMBER (MITDB)	AF Data Record Number (AFDB)	Normal Data Record Number (NSRDB)
1	100	4015	16,265
2	101	4043	16,272
3	102	4048	16,273
4	103	4126	16,420
5	104	4746	16,483
6	105	4908	16,539
7	106	4936	16,773
8	107	5091	16,786
9	108	5121	16,795
10	109	5261	17,052
11	111	6426	17,453
12	112	6453	18,177
13	113	6995	18,184
14	114	7162	19,088
15	115	7859	19,090
16	116	7879	19,093
17	117	7910	19,140
18	118	8215	19,830
19	119	8219	
20	121	8378	

21	122	8405
22	123	8434
23	124	8455
24	200	
25	201	
26	202	
27	203	
28	205	
29	207	
30	208	
31	209	
32	210	
33	212	
34	213	
35	214	
36	215	
37	217	
38	219	
39	220	
40	221	
41	222	
42	223	
43	228	
44	230	
45	231	
46	232	
47	233	
48	234	

1. Moody GB, Mark RG. The impact of the MIT-BIH arrhythmia database. IEEE Engineering in Medicine and Biology Magazine. 2001;20(3):45–50.
