# Supplementary figures and images for: An improved method to detect arrhythmia using ensemble learning-based model in multi lead electrocardiogram (ECG)
Source: PLoS One. 2024 Apr 9;19(4):e0297551. doi: 10.1371/journal.pone.0297551 (PMC11003640; doi:10.1371/journal.pone.0297551)

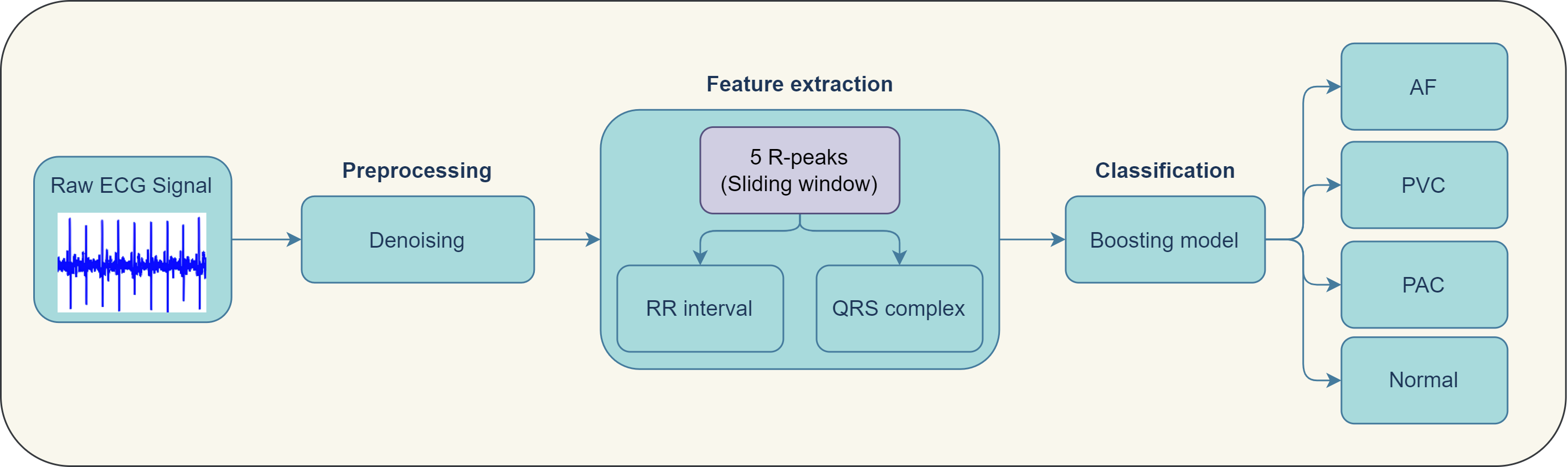

Supplement: S1 Graphical abstract — (PNG) [file pone.0297551.s002.png]
